# Exploiting a subtype-specific mitochondrial vulnerability for successful treatment of colorectal peritoneal metastases

**DOI:** 10.1016/j.xcrm.2024.101523

**Published:** 2024-04-25

**Authors:** Sanne Bootsma, Mark P.G. Dings, Job Kesselaar, Roxan F.C.P.A. Helderman, Kyah van Megesen, Alexander Constantinides, Leandro Ferreira Moreno, Ellen Stelloo, Enzo M. Scutigliani, Bella Bokan, Arezo Torang, Sander R. van Hooff, Danny A. Zwijnenburg, Valérie M. Wouters, Vincent C.J. van de Vlasakker, Laskarina J.K. Galanos, Lisanne E. Nijman, Adrian Logiantara, Veronique L. Veenstra, Sophie Schlingemann, Sterre van Piggelen, Nicole van der Wel, Przemek M. Krawczyk, Johannes J. Platteeuw, Jurriaan B. Tuynman, Ignace H. de Hingh, Jan P.G. Klomp, Arthur Oubrie, Petur Snaebjornsson, Jan Paul Medema, Arlene L. Oei, Onno Kranenburg, Clara C. Elbers, Kristiaan J. Lenos, Louis Vermeulen, Maarten F. Bijlsma

**Affiliations:** 1 Amsterdam UMC Location University of Amsterdam, Center for Experimental and Molecular Medicine, Laboratory of Experimental Oncology and Radiobiology, Amsterdam, the Netherlands; 2 Oncode Institute, Amsterdam, the Netherlands; 3 Cancer Center Amsterdam, Cancer Biology, Amsterdam, the Netherlands; 4 Amsterdam Gastroenterology Endocrinology Metabolism, Amsterdam, the Netherlands; 5 Laboratory of Translational Oncology, UMC Utrecht Cancer Center, Utrecht, the Netherlands; 6 Department of Genetics, Center for Molecular Medicine, University Medical Center Utrecht, Utrecht University, Utrecht, the Netherlands; 7 Amsterdam UMC Location University of Amsterdam, Department of Medical Biology, Amsterdam, the Netherlands; 8 Amsterdam UMC Location University of Amsterdam, Department of Radiation Oncology, Amsterdam, the Netherlands; 9 Amsterdam UMC Location University of Amsterdam, Electron Microscopy Center, Amsterdam, the Netherlands; 10 Avivia Projects BV, Boxtel, the Netherlands; 11 Amsterdam UMC Location Vrije Universiteit Amsterdam, Department of Surgery, Cancer Center Amsterdam, De Boelelaan 1117, Amsterdam, the Netherlands; 12 Department of Surgery, Catharina Hospital, Eindhoven, the Netherlands; 13 GROW – School for Oncology and Developmental Biology, Maastricht University, Maastricht, the Netherlands; 14 Lead Pharma, Oss, the Netherlands; 15 Department of Pathology, The Netherlands Cancer Institute, Amsterdam, the Netherlands

**Keywords:** peritoneal metastases, oxidative phosphorylation, mitochondria, elesclomol, copper, HIPEC, molecular subtype, mesenchymal cancer cell

## Abstract

Peritoneal metastases (PMs) from colorectal cancer (CRC) respond poorly to treatment and are associated with unfavorable prognosis. For example, the addition of hyperthermic intraperitoneal chemotherapy (HIPEC) to cytoreductive surgery in resectable patients shows limited benefit, and novel treatments are urgently needed. The majority of CRC-PMs represent the CMS4 molecular subtype of CRC, and here we queried the vulnerabilities of this subtype in pharmacogenomic databases to identify novel therapies. This reveals the copper ionophore elesclomol (ES) as highly effective against CRC-PMs. ES exhibits rapid cytotoxicity against CMS4 cells by targeting mitochondria. We find that a markedly reduced mitochondrial content in CMS4 cells explains their vulnerability to ES. ES demonstrates efficacy in preclinical models of PMs, including CRC-PMs and ovarian cancer organoids, mouse models, and a HIPEC rat model of PMs. The above proposes ES as a promising candidate for the local treatment of CRC-PMs, with broader implications for other PM-prone cancers.

## Highlights

• CMS4 subtype explains lack of efficacy of current HIPEC treatment in CRC-PMs
• CMS4 cells have reduced numbers of mitochondria
• Elesclomol targets oxidative phosphorylation in unique fashion
• Elesclomol is effective in preclinical PM models

## Graphical abstract

**Figure:**
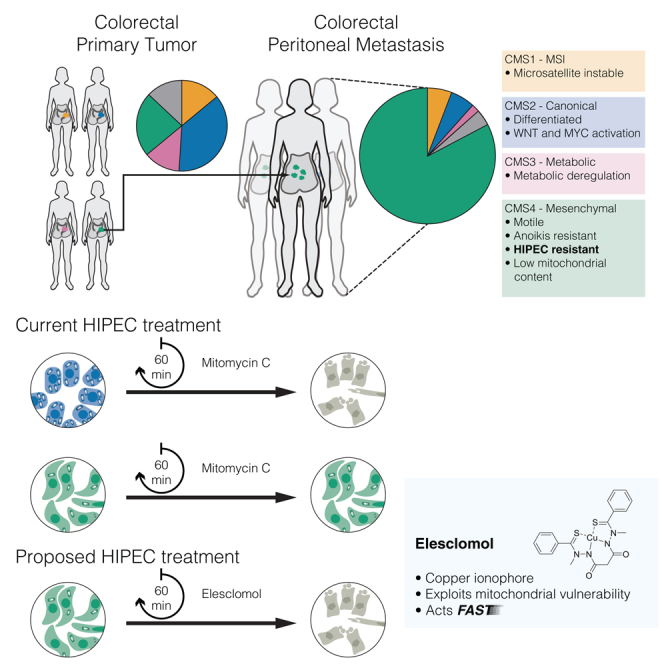

## Teaser

Bootsma et al. reveal that CMS4-subtype colorectal peritoneal metastases show a dismal prognosis despite surgery with local chemotherapy. Elesclomol was identified as a potent new compound, demonstrating selective cytotoxicity against CMS4 cells by targeting oxidative phosphorylation machinery. Elesclomol proves effective in preclinical peritoneal metastases models, suggesting its potential for local treatment.

## Introduction

Peritoneal metastases (PMs) in colorectal cancer (CRC) are associated with a dismal prognosis and a high burden of disease. Synchronous PMs occur in 4.3%–7.7% of all patients with CRC, with a median overall survival of only 8 months^1^^,^^2^^,^^3^^,^^4^ Additionally, metachronous PMs are seen in about 5% of CRC patients.^1^^,^^2^^,^^3^ Although peritoneal metastatic disease (PMD) can be accompanied by more widespread metastases, it often occurs as the only sign of dissemination.^5^ This implies that the route of metastatic spread to the peritoneum differs from that to distant organs. The metastatic cascade that leads to PM formation places specific demands on the cancer cells, such as the ability to survive in a nutrient-deprived environment, and specific molecular features of cancers involving the peritoneum have been described.^6^

We and others have previously found that CRC-PMs almost universally classify as consensus molecular subtype 4 (CMS4).^7^^,^^8^ This previously recognized disease entity is characterized by mesenchymal features and poor prognosis.^9^ Furthermore, CMS4 is associated with resistance to currently used therapies, including oxaliplatin.^10^^,^^11^ Cytoreductive surgery with hyperthermic intraperitoneal chemotherapy (CRS-HIPEC) is the only treatment option with curative intent for CRC-PM patients with limited disease. The randomized PRODIGE 7 trial did, however, not report a survival advantage of CRS-HIPEC with oxaliplatin over CRS alone.^12^ In addition, lack of therapeutic effect of both mitomycin C (MMC) and oxaliplatin is demonstrated by the high recurrence rates of PMs after complete CRS and HIPEC.^13^^,^^14^ This could be explained by the resistance of the CMS4 subtype for the used chemotherapeutics. Thus, novel treatment strategies that successfully target the CMS4 subtype are needed.

Large-scale compound screens in cancer cell lines are valuable tools for drug discovery and repurposing efforts. In this report, we identify the copper (Cu) ionophore elesclomol (ES) as a highly effective agent against CMS4-specific disease models. ES kills cancer cells in a Cu-dependent fashion by targeting the oxidative phosphorylation machinery, which we found to be a specific vulnerability of CMS4 cancers. ES is effective following only minutes of exposure, supporting its use in intra-abdominal treatment procedures. It is therefore a promising candidate for the local treatment of CRC-PMs.

## Results

### PMs are a manifestation of mesenchymal-subtype CRC

**Figure 1:**
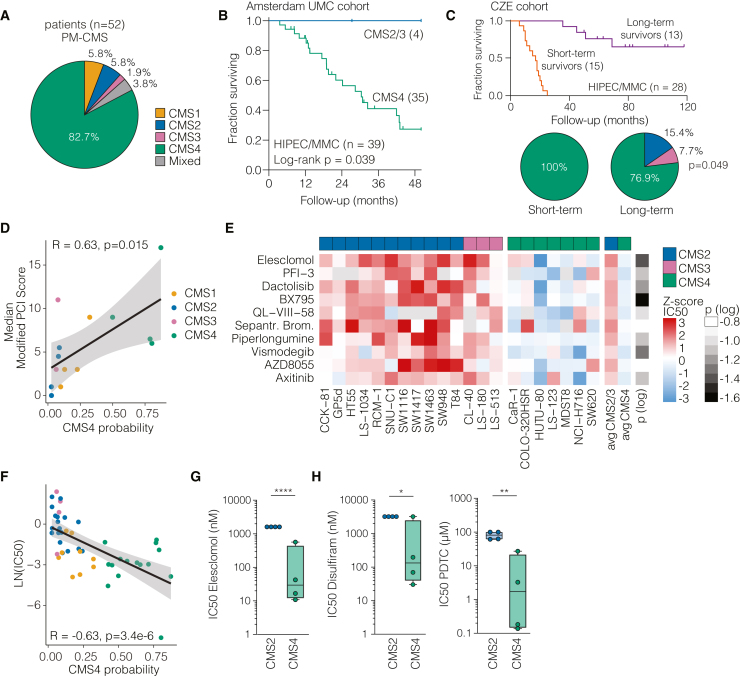
Characteristics and vulnerabilities of CMS4 CRC cancer and PMs (A) Distribution of CMS subtypes among patients with CRC-PMs (*n* = 52) (GEO: GSE183202). (B) Kaplan-Meier plot of OS of patients in the Amsterdam UMC cohort treated with CRS-HIPEC with MMC, stratified by CMS2/3 (blue) and CMS4 (green). The x axis represents time in months since the CRS-HIPEC procedure. The *p* value was obtained using the log rank test. (C) Kaplan-Meier plot of OS of patients in the Catharina Ziekenhuis Eindhoven (CZE) cohort, all treated with CRS-HIPEC with MMC. Patients are stratified by short-term survival (OS < 24 months, *n* = 15) or long-term survival (OS > 36 months, *n* = 13). The x axis represents the time in months since the CRS-HIPEC procedure. Pie charts show distribution of CMS by survival groups. Statistical analysis was performed using chi-square test between CMS2/3 and CMS4. (D) Correlation between CMS4 probability of 15 CRC cell lines and peritoneal engraftment, scored as mPCI, in immunodeficient mice. The PCI score is an average of minimally 3 mice per cell line. Statistical analysis was performed using Pearson correlation. (E) Heatmap showing the GDSC’s *Z* score of drug sensitivity (IC50) in CRC cell lines for the indicated compounds. Compounds are ranked by Δ*Z* score CMS2/3 vs. CMS4. Statistical analysis was performed using unpaired t test. (F) IC50 values reported in the GDSC database (natural log transformed) for ES for the cell lines shown in (D) were correlated to the CMS4 probability of the cell lines. Statistical analysis was performed using Pearson correlation. (G) IC50 values of CMS2 (*n* = 4) and CMS4 (*n* = 4) cell lines treated with ES for 72 h. Data are means ± SD, minimally 3 biological replicates per cell line. When IC50 was not reached, the highest tested concentration was plotted. Statistical analysis was performed using unpaired t test. ∗*p* ≤ 0.05, ∗∗*p* ≤ 0.01, ∗∗∗∗*p* ≤ 0.0001. (H) As for (F), using disulfiram and PDTC.

Recently, we generated transcriptomic profiles of 82 samples from 52 CRC-PM patients and found that the vast majority of patients (82.7%, 43 of 52) were classified as CMS4 (Figure 1A).^7^ This is a marked overrepresentation compared to studies encompassing all stages of CRC, where CMS4 typically accounts for ∼25% of cases.^9^ Additionally, in studies specifically focusing on liver metastasis, the percentage of CMS4 does not exceed 35%.^15^^,^^16^ Furthermore, in our cohort, we classified three patients as CMS1, three as CMS2, and one as CMS3, each occurring at much lower frequency compared to primary cancer cohorts.^7^^,^^9^

In addition to bearing increased invasive capacities, CMS4 cells are also considered more resistant to currently applied (chemo)therapeutics.^10^^,^^11^ A subset of patients in our cohort was treated with CRS-HIPEC using MMC. Whereas this proved to be an effective treatment in the relatively few patients with CMS2/3 PMD, a high proportion of CMS4 patients presented with recurrence and finally succumbed to the disease (Figures 1B and S1A). The peritoneal cancer index (PCI) and resection score did not differ between the groups (Table S1). These data indicate that current PM-directed treatments are not effective in the majority of patients, as they present with a distinct, resistant molecular subtype.

To establish orthogonal evidence, we generated a new RNA sequencing (RNA-seq) dataset from 28 CRC-PM patients who received CRS-HIPEC with MMC. Patients were selected for short (<24 months) or long (>36 months) overall survival (OS) after CRS-HIPEC (Figure 1C). No differences in gender, age, PCI, or resection score were found between the groups (Table S2). CMS classification of the cohort revealed an enrichment of CMS2/3 in the long survival group (0% versus 23.1%, chi-square test, *p* = 0.049). These data confirm that CMS4 CRC-PM patients are resistant to currently used chemotherapeutics.

Next, we ascertained that the ability to grow as PMs is a CMS4 cell-intrinsic feature. We used a collection of cell lines that we previously used to establish an *in vivo* model of PMs, which were assigned continuous CMS4 probability scores.^17^ Following injection of these cell lines into the peritoneal cavity of immune-compromised (nude) mice, tumor burden was quantified using the modified peritoneal cancer index (mPCI) score. Peritoneal tumor burden was highly correlated to the CMS4 probability of CRC cell lines (Figure 1D). These analyses and previous work from our group establish that the mesenchymal CMS4 subtype is strongly associated with PMs and that cell lines can capture this CMS-specific feature in a tractable system with high fidelity. The accuracy with which CMS4 cell lines recapitulate key characteristics of this disease suggest that they may be used to identify effective treatments for CRC-PMs.

### ES is effective against CMS4 cancer cells

To identify compounds with specific activity against CMS4 cancers, we analyzed drug sensitivity data from the Genomics of Drug Sensitivity in Cancer (GDSC) database.^18^ We compared the drug sensitivity profiles of CMS4 cell lines to other microsatellite stable subtypes (CMS2/3) in CRC cell lines. Among the top 10 compounds ranked by Δ*Z* score of mean half maximal inhibitory concentration (IC50) (CMS2/3 versus CMS4 cell lines), ES was the top hit (Figure 1E). The IC50 values of ES were indeed strongly negatively correlated with the CMS4 probability scores of CRC cell lines (Figure 1F). The specificity of ES against CMS4 cells could be independently validated (Figure 1G). Notably, our investigation extended beyond CRC, as cell lines representing a mesenchymal subtype in PM-prone pancreatic ductal adenocarcinoma (PDAC)^19^^,^^20^ displayed greater sensitivity to ES compared to non-mesenchymal cell lines (Figures S1B and S1C). Previous work has shown that ES is a Cu ionophore.^21^^,^^22^ The Cu ionophores disulfiram and pyrrolidine dithiocarbamate (PDTC), which also harbor Cu-shuttling activity but were not included in the GDSC database, showed the same differential efficacy against CMS4 cells as compared to CMS2 cells (Figure 1H). This suggests that mesenchymal-subtype cancer cells possess a particular vulnerability to Cu-induced cell death.

### ES-Cu disrupts mitochondrial function

**Figure 2:**
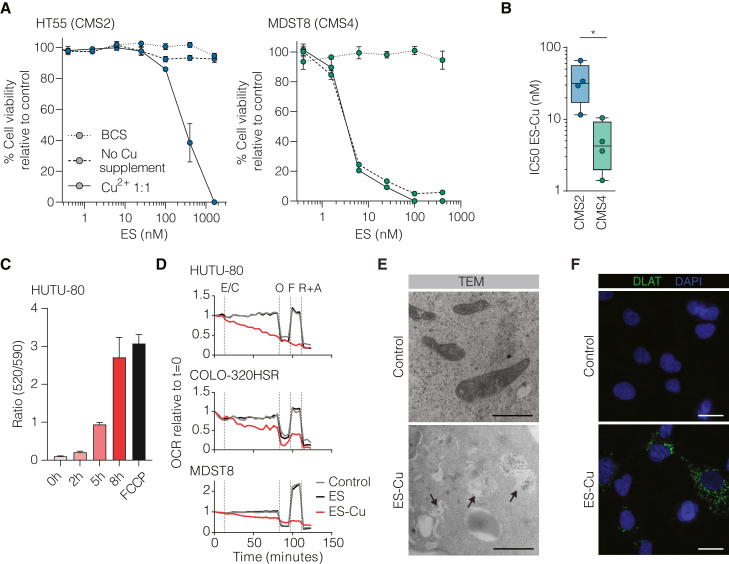
ES is a Cu ionophore and disrupts mitochondrial function (A) Dose-response curves of CMS2 and CMS4 cell lines treated with ES, ES with CuCl_2_ at a 1:1 ratio, or ES with 200 μM BCS; readout after 72 h. Data are means ± SD of 3 biological replicates, normalized to solvent control. Medium with serum (but with no supplemented Cu) contains 0.18 μM Cu^2+^. (B) IC50 values of CMS2 (*n* = 4) and CMS4 (*n* = 4) cell lines treated with ES + 5 μM CuCl_2_ for 72 h. Data are means ± SD, minimally 3 biological replicates per cell line. Statistical analysis was performed using unpaired t test. ∗*p* ≤ 0.05. (C) Mitochondrial depolarization measured by JC-10 (mean fluorescence of monomer/aggregate). The samples were treated with ES for 0, 2, 5, and 8 h or with trifluoromethoxy carbonylcyanide phenylhydrazone (FCCP) for 15 min. Data are means ± SD of 3 biological replicates. (D) Seahorse flux analysis of the indicated cells to measure the real-time oxygen consumption rate (OCR). 5 μM ES was added at t = 12 min by injection into the analyzer. Under the control and ES-Cu conditions, 5 μM CuCl_2_ was present at all times. A regular Mito Stress Test was applied: oligomycin to inhibit ATP synthesis, FCCP to uncouple the mitochondria, and rotenone/antimycin A to block the mitochondrial electron transport chain. Compounds were added at the indicated times. Mean values of minimally 6 technical replicates are normalized to t = 0. E/C, ES with or without CuCl_2_; O, oligomycin; F, FCCP; R+A, rotenone with antimycin A. (E) Transmission electron microscopy images from HUTU-80 cells that were exposed to control or ES-Cu treatment, 5 μM ES-Cu for 2 h, after which cells were fixed and imaged. Scale bars, 2 μm. Arrows indicate damaged mitochondria. (F) Immunofluorescence images from HUTU-80 cells that were exposed to control or ES-Cu treatment, stained with anti-DLAT antibody to visualize DLAT aggregates. 500 nM ES-Cu for 30 min, imaging 24 h after treatment. Scale bars, 25 μm.

Next, we investigated whether combined treatment of cancer cells with ES and Cu enhanced anti-cancer cell efficacy. Indeed, equimolar addition of Cu to ES increased efficacy against the CMS2 cell line HT55 (Figure 2A). For CMS4 cells, Cu supplementation in addition to the Cu already present in the serum-containing culture medium did not lead to increased sensitivity. Notably, addition of the Cu chelator bathocuproinedisulfonic acid (BCS) rescued the cells from the cytotoxic effect of ES. In addition, Cu supplementation without ES was not toxic at this concentration (Figures S2A and S2B). We confirmed the influx of Cu into the cells using ICP-MS analysis after ES alone or ES with the addition of Cu (ES-Cu) treatment (Figure S2C). The difference between CMS2 and CMS4 cell line sensitivity was retained when treating the cells with equimolar ES-Cu (Figure 2B). This suggests that the differential sensitivity cannot be attributed to differences in baseline Cu levels or influx of Cu.

ES-Cu has been identified as a potent reactive oxygen species (ROS)-generating agent due to the introduction of highly chemically reactive Cu^2+^ into the mitochondria, ultimately leading to apoptosis.^23^ Caspase activity measurements confirmed activation of apoptotic programs (Figure S2D). Furthermore, ES-Cu treatment triggered the production of mitochondrial ROS prior to cell death (Figure S2E). However, the addition of antioxidants failed to rescue the cells, indicating that the cytotoxic effect of ES occurred before excessive ROS production (Figure S2F).

To better understand the impact of ES treatment on mitochondrial metabolism, we examined the mitochondrial membrane potential and oxygen consumption rate shortly after adding ES-Cu. We found that the mitochondrial membrane potential was lost (Figure 2C), and oxygen consumption was completely impaired within minutes of addition (Figure 2D). Importantly, this loss of oxygen consumption was dependent on the presence of Cu, as treatment with ES alone or Cu alone (Figure S2G) did not impact oxygen consumption. Furthermore, transmission electron microscopy revealed that treatment with ES-Cu induced swelling and disruption of the mitochondrial structure, which could explain why antioxidants were ineffective in rescuing the cells (Figure 2E). The severe mitochondrial disruption we observed is not a known morphological characteristic of known cell death pathways (apoptosis, ferroptosis, necrosis, and autophagy).^24^

To further investigate the link between mitochondrial metabolism and ES sensitivity, we replaced glucose with galactose in the medium to induce an oxidative phosphorylation-reliant state in CRC cell lines. This resulted in an almost 2.5-fold increase in sensitivity to ES-Cu in both CMS2 and CMS4 cells (Figure S2H). Recent studies have shown that ES-Cu perturbs mitochondrial iron-sulfur (Fe-S) complexes, leading to the aggregation of lipoylated proteins such as dihydrolipoamide *S*-acetyltransferase (DLAT) in mitochondria and the emergence of a novel form of cell death known as cuproptosis.^22^ We observed the appearance of DLAT clusters following exposure to 500 nM ES-Cu, confirming the involvement of cuproptosis in ES-induced cell death (Figure 2F). Taken together, our results demonstrate that ES-Cu triggers a form of cell death that is heavily dependent on mitochondrial function.

### A paucity of mitochondria in CMS4 cancer cells correlates with sensitivity to ES-Cu

**Figure 3:**
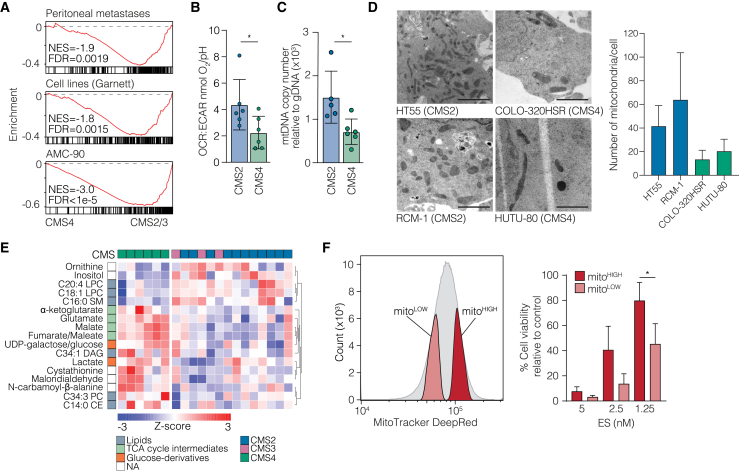
Reduced mitochondrial content in CMS4 cells explains sensitivity to ES (A) Gene set mRNA enrichment analysis depicting the Hallmark Oxidative Phosphorylation signature, comparing CMS4 vs. CMS2/3 in CRC-PM patient samples (*n* = 47), CRC cell lines (*n* = 37), and primary CRC patient samples from the AMC90 dataset (*n* = 71). NES, normalized enrichment score; FDR, false discovery rate. (B) Results from Seahorse analysis measuring the baseline ratio of OCR:extracellular acidification rate in 12 cell lines, grouped by subtype. The bar graph shows mean ± SD of minimally 6 technical replicates, and each dot represents an independent cell line. Statistical analysis was performed using unpaired t test. ∗*p* ≤ 0.05. (C) Mitochondrial abundance, quantified by mtDNA copy number in 11 cell lines, grouped by subtype. The bar graph shows mean ± SD of 3 technical replicates, and each dot represents an independent cell line. Statistical analysis was performed using unpaired t test. ∗*p* ≤ 0.05. (D) Representative transmission electron microscopy images (scale bars, 2 μm) of the HT55, RCM-1, HUTU-80, and COLO-320HSR (respectively, 2 CMS2 and 2 CMS4) cell lines. The bar graph shows mean ± SD of the number of mitochondria counted per cell from a single experiment. 23–26 cells per cell line were counted using RADIUS quantification software. Unpaired t test, pooled CMS2 vs. CMS4 cells, *p* < 0.0001. (E) Heatmap depicting the most differential metabolites in CMS2/3 (*n* = 14) vs. CMS4 (*n* = 6) cell lines using metabolomics data from the Cancer Cell Line Encyclopedia (CCLE) metabolomics cell line database.^26^ (F) Histogram of sorted populations stained with MitoTracker DeepRed (mitochondria [mito]^LOW^ and mito^HIGH^) and corresponding bar graphs depicting the cell viability 72 h after treatment with the indicated concentrations of ES in sorted populations using HUTU-80 cells. Data are means ± SD of 3 biological replicates. Statistical analysis was performed using unpaired t test. ∗*p* ≤ 0.05.

To chart the cell biology that may explain the specific efficacy of ES against CMS4 cells, we assessed expression data from PM lesions, cell lines, and primary tumors.^7^^,^^18^^,^^25^ This revealed that, compared to CMS2/3, gene sets involved in oxidative phosphorylation were among the top downregulated signatures both *in vitro* and in patients (Figure 3A; Table S3). Likewise, when comparing CMS4 cell lines to the other microsatellite stable subtypes (CMS2/3), CMS4 lines were found to express low levels of nuclear gene transcripts coding for proteins across all oxidative phosphorylation complexes (Figure S3A). Flux analysis showed lower oxygen consumption by mesenchymal subtype cells (CRC, Figure 3B; PDAC, Figure S3B), and this was supported by a strongly reduced mitochondrial content in CMS4 cells, revealed by mtDNA copy number measurements and transmission electron microscopy (Figures 3C and 3D). Publicly available metabolomics data^26^ for CMS-classified CRC cells revealed elevated levels of lactate and tricarboxylic acid (TCA) cycle intermediates in CMS4 cells, supporting a reduced oxidative phosphorylation capacity (Figures 3E and S3C). In Clinical Proteomic Tumor Analysis Consortium (CPTAC) proteomics data,^27^ we found that mitochondrial markers commonly used in immunohistochemistry, COX4I1 (localized on the mitochondrial inner membrane) and TOMM20 (localized on the mitochondrial outer membrane), were upregulated in CMS2/3, as was the key activator of mitochondrial transcription, mitochondrial transcription factor A (TFAM) (Figure S3D). In line with this, lactate dehydrogenase A (LDHA) was slightly increased in CMS4. This supports that lower mitochondrial content in CMS4 can also be detected in patient samples.

To establish a causal relationship between mitochondrial content and ES sensitivity, we inhibited mitochondrial biogenesis by pharmacologically targeting the transcription factor estrogen-related receptor alpha (ESRRA) in the HT55 CMS2 cell line, which has high mitochondrial content and is normally relatively resistant to ES.^28^^,^^29^ Inhibition of ESRRA resulted in a sensitizing effect for cell killing by ES (Figures S3E‒S3G). To mitigate the risk that this is caused by off-target effects of the ESRRA inhibitor, we used an isogenic setup in which we sorted CMS4 cells for mitochondrial content using MitoTracker DeepRed (Figure 3F) or pLV-mitoDsRed (Figure S3H). Indeed, cells with low mitochondrial content were most sensitive to ES. Thus, it appears that disruption of mitochondria by ES (and other Cu ionophores, such as disulfiram) is effective against mesenchymal subtype cancer cells due to their low mitochondrial content.

### Short-term exposure to Cu ionophores suffices for effective cancer cell killing

**Figure 4:**
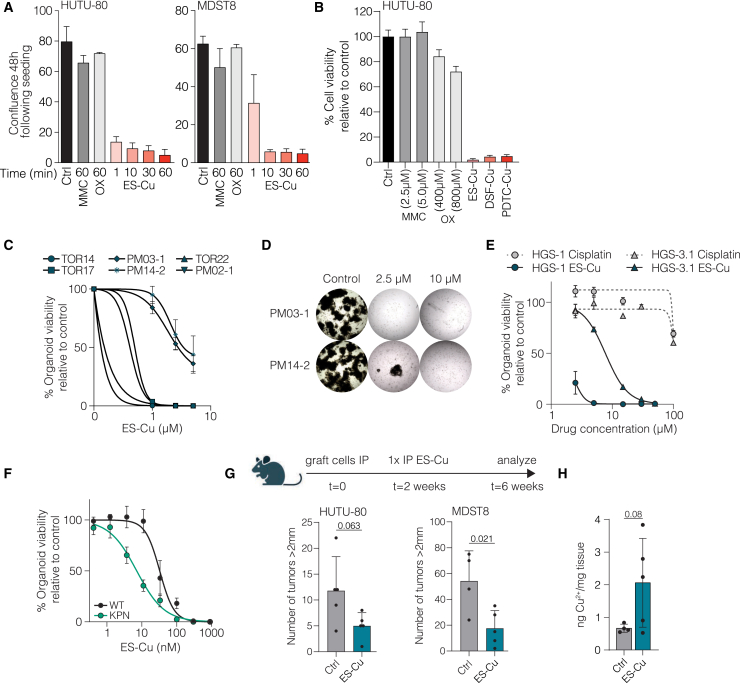
The mechanism of action of ES allows rapid cell killing (A) Cells were exposed to 1, 10, 30, and 60 min of ES-Cu (2.5 μM), 60 min MMC (2.5 μM), or 60 min oxaliplatin (OX; 400 μM). After washing, cells were left to grow for 48 h, after which confluence was assessed by IncuCyte microscopy. Data are means ± SD of minimally 3 biological replicates. (B) Cells were exposed to 10 min of MMC (2.5 and 5 μM), OX (400 and 800 μM), ES-Cu (2.5 μM), DSF-Cu, (5 μM), or PDTC-Cu (10 μM). After washing, cells were left to grow for 48 h, after which cell viability was assessed by CellTiter Blue. Data are means ± SD of 3 biological replicates. (C) A panel of PM-derived organoids was exposed to 60-min treatment with ES-Cu. After washing, cells were left to grow for 6 days, after which cell viability was assessed by CellTiter-Glo. Data are means ± SD of 4 biological replicates; lines are fitted from the data points. (D) Long-term outgrowth potential of ascites-derived organoids was determined. Organoids were incubated with ES-Cu for 60 min at the indicated concentrations. Images were taken 24 days after treatment. Representative images are shown. The diameter of the images is 3.0 mm. (E) Cell viability from pulse-treated, PM-derived ovarian organoids. Organoids were exposed to ES-Cu or cisplatin for 60 min. After washing, organoids were left to grow for 6 days, after which cell viability was assessed by CellTiter-Glo. Dots represent mean ± SD from 3 biological replicates; lines are fitted from the data points. (F) Cell viability from murine WT organoids and KPN tumoroids. Cells were exposed to ES-Cu for 72 h. Cell viability was assessed by CellTiter-Glo. Dots represent mean ± SD from 3 biological replicates; lines are fitted from the data points. (G) Immune-deficient mice were grafted with HUTU-80 or MDST8 cells. After 2 weeks, mice received a single intraperitoneal 1-mL injection of vehicle control or 25 μM ES-Cu. After an additional 4 weeks, mice were sacrificed, and tumor burden was assessed. Quantification of the number of tumors > 2 mm was performed, *n* = 4–5 mice per group. Data are means ± SD. Statistical analysis was performed using unpaired t test. The mouse image was created with BioRender. (H) Cu levels were determined by ICP-MS in MDST8 peritoneal tumors collected 3 h after treatment with vehicle or 2.5 μM ES-Cu (*n* = 4–5 tumors per group). Data are means ± SD.

HIPEC procedures for the treatment of PMs involve infusion of chemotherapy in the abdominal cavity for 60–120 min, after which the chemotherapy is removed. This requires that agents used in these procedures act fast. Given the profound effects on mitochondria observed after relatively short exposure, we reasoned that ES-Cu may harbor the desired rapid activity against cancer cells. We evaluated the anti-cancer effects of various exposure times to ES-Cu and the cytotoxic agents currently in use for HIPEC. Remarkably, near-complete cell growth inhibition was achieved after only 10-min of exposure (Figure 4A). This sensitivity following short exposure times was also observed after 10-min treatment with the Cu ionophores disulfiram and PDTC, in strong contrast with the marked inefficacy of high doses of MMC and oxaliplatin in the time frames tested (Figure 4B). The applied concentrations of MMC and oxaliplatin were based on concentrations found in HIPEC perfusate^30^ and were highly similar to mean IC50 values reported in the GDSC (Figures S4A and S4B). This supports the potential of ES-Cu as a candidate for application in intra-abdominal therapies.

### Efficacy of ES in preclinical models for PMD

To further demonstrate the efficacy of ES-Cu against PMD, we evaluated its effects on organoid cultures directly derived from peritoneal tumor depositions from patients with CRC (TOR14, TOR17, and TOR22) or tumor cells in ascites fluid from CRC-PM patients (PM02-1, PM03-1, and PM14-2) (Table S4).^8^^,^^30^ ES-Cu was effective in reducing viability in this extensive panel of CRC-PM organoids after a 60-min washout treatment (Figure 4C).

Notably, ascites-derived PM03-1 and PM14-2 were not completely killed by the used concentration and time frame. Therefore, we performed an outgrowth assay in which organoids were left to grow out for an additional 21 days. This revealed that ES-Cu was uniformly effective against PM organoids irrespective of derivation site (Figure 4D). Additionally, considering that ovarian cancer leads to PMs in the majority of patients,^31^ we also treated patient-derived organoids from ovarian cancer with ES-Cu for 60 min. ES-Cu outperformed cisplatin, the current HIPEC treatment for ovarian cancer (Figure 4E).^32^ To assess whether a therapeutic window exists to target cancer cells but not healthy tissue, we tested ES-Cu in wild-type (WT) intestinal organoids^33^ and genetically engineered CMS4 tumoroids (*VillinCre*^*ER*^ *Kras*^*G12D/+*^ *Trp53* ^*fl/fl*^ *Rosa26*^*N1icd/+*^; KPN).^34^ The CMS4 tumoroids were almost 5 times more sensitive to ES-Cu compared to WT organoids (IC50: 32.6 nM and 7.1 nM, respectively; Figure 4F). Altogether, the results confirm that ES-Cu holds promise to replace classical cytostatic drugs in a HIPEC treatment setting.

To evaluate the efficacy of ES-Cu *in vivo*, we utilized our PM mouse model, where CMS4 tumor cells are injected intraperitoneally into immunodeficient mice.^17^ Two weeks after tumor cell injection, mice were intraperitoneally injected with a relatively high volume (1 mL) of 25 μM ES-Cu solution (Figure 4G). After an additional 4 weeks, mice were sacrificed, and tumor load was quantified. Treatment with ES-Cu led to a statistically significant reduction in mice bearing MDST8 peritoneal tumors but not to complete eradication (Figures 4G and S4C). Higher or more frequent dosing led to toxicity in the mice (data not shown). Analysis of Cu levels in tumors collected 3 h after treatment demonstrated a marginally significant increase in Cu levels in part of the tumors upon ES-Cu administration (Figure 4H). The high standard error in these measurements could be explained by uneven ES-Cu distribution in the peritoneal cavity. Additionally, the observed levels may not be sufficient to achieve complete tumor cell eradication.

**Figure 5:**
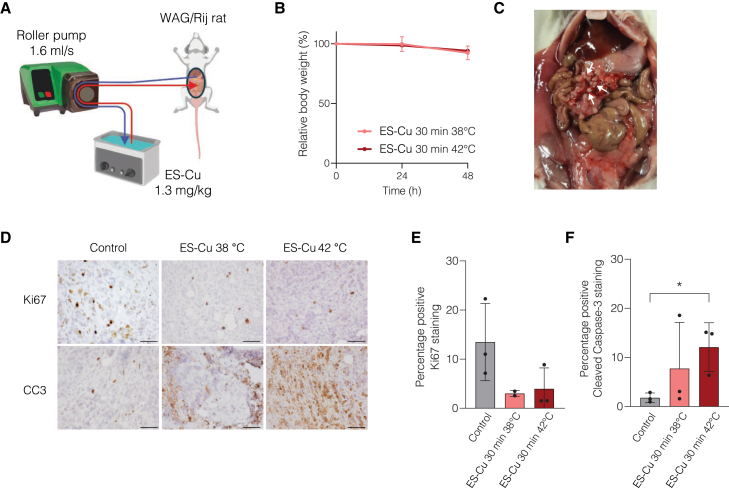
ES-Cu is safe in a rat HIPEC model and impacts tumor cell viability (A) Schematic overview of the rat HIPEC setup, adapted from Löke et al.^35^ A total of 500 mL ES-Cu solution (1.3 mg/m^2^) was heated to 38°C or 42°C in a water bath, circulated by a roller pump (1.6 mL/s), and administered intraperitoneally via a semi-open in/outflow construction containing four perforated inflow tubes and one outflow catheter. Treatment duration was 30 min. (B) Relative body weight, measured every 24 h, remains stable after the HIPEC procedure in ES-Cu-treated rats. Data are means ± SD of 3 rats per condition. (C) Photo of intraperitoneal tumor lesions in a rat, 16 days after intraperitoneal injection with CC531 cells. Arrows indicate tumor lesions. (D) Representative images of Ki67 and cleaved caspase (CC3) immunohistochemistry on tumors collected 48 h after HIPEC or no treatment. Scale bars, 50 μm. (E) Quantification of Ki67 staining performed in (D). Dots are means of tumors in 3 regions (liver, stomach, and omentum), bars indicate ±SD of 3 rats per condition. (F) Quantification of CC3 staining performed in (D). Dots are means of tumors in 3 regions (liver, stomach, and omentum), bars indicate ±SD of 3 rats per condition. Statistical analysis was performed using unpaired t test. ∗*p* ≤ 0.05.

We reasoned that the parameters unique to HIPEC (high perfusate volume, adequate perfusate distribution, and high temperature) could enhance the efficacy of ES-Cu. Treating CMS4 cells at 42°C indeed led to increased sensitivity to ES-Cu (Figure S5A). To further evaluate the therapeutic potential of ES-Cu in a HIPEC setting, we assessed the efficacy of ES-Cu in a rat model that allows HIPEC, recently developed in our group (Figure 5A).^35^ In short, rats were intraperitoneally injected with the syngeneic CRC cell line CC531, which is classified as CMS4^36^ and is sensitive to ES-Cu long-term (Figure S5B) and short-term (Figure S5C) treatment. Ultrasound monitoring was performed until tumors reached a size of 4–6 mm. Subsequently, a heated solution of ES-Cu (38°C or 42°C) was perfused through the peritoneal cavity for 30 min. Afterward, the abdomen was closed again. Of note, CC531 cells are not sensitive to heat alone in this time frame.^36^ The procedure was well tolerated and did not lead to weight loss or additional observed discomfort (Figure 5B). After 48 h, rats were sacrificed, and tumor lesions were collected (Figure 5C). Immunohistochemical staining of Ki67 hinted that ES-Cu treatment inhibited tumor cell proliferation (Figures 5D and 5E). Also, ES-Cu combined with hyperthermia effectively induced apoptosis (Figures 5D and 5F). Collectively, these findings further support the potential of ES-Cu as a promising drug for the local treatment of PMD.

## Discussion

PMD in CRC poses a significant challenge due to the association with a dismal prognosis and a high burden of disease. PM lesions are almost universally classified as CMS4, a subtype of CRC already associated with poor prognosis and therapy resistance. No curative treatments are available for the majority of patients with PMD. In this report, we demonstrate that patients with CMS4 PM indeed have poor survival outcomes after CRS-HIPEC therapy.^7^ We set out to identify new therapeutic compounds with improved pharmacodynamics that are suitable for local treatment in a HIPEC setting; i.e., cytotoxic after a short exposure. ES, a Cu ionophore, was found to be most effective in CMS4 cancer cells and highly potent after short exposure times. Other Cu ionophores showed similar traits, which suggest this class of molecules to be promising for future HIPEC treatments. In addition, we found that CMS4 cells are particularly vulnerable for this class of drugs because of their decreased mitochondrial respiration due to reduced numbers of mitochondria.

ES was initially developed by Synta Pharmaceuticals as an agent against melanoma.^23^ A phase 3 combination clinical trial of ES with paclitaxel in patients with melanoma showed a lack of efficacy.^37^ However, no patient stratification was performed in this trial. We propose that specific subtypes of tumors (for instance, the mesenchymal CMS4 subtype in CRC), are in fact vulnerable to ES treatment. Furthermore, no additional Cu was used in the trial, and treatment was given systemically. Local intraperitoneal treatment with the addition of Cu may represent a promising new application for the clinical use of ES. Interestingly, clinical trials are being performed to investigate the benefit of intraperitoneal administration of chemotherapy to complement the systemic chemotherapy.^38^ It is thought that, with intraperitoneal administration, higher peak concentrations can be reached, resulting in enhanced efficacy while minimizing systemic side effects. The clinical translation of ES and the way it should be administered remain topics of future research.

The mechanism of action of ES relies on the shuttling of Cu to the mitochondria. It has been proposed that low ES concentrations trigger a new cell death pathway referred to as cuproptosis.^22^ The transported Cu binds to components of post-translational modifications called lipoylation. Lipoylation only occurs in a few metabolism-related enzymes, most of them located in the mitochondria. Direct binding of Cu to lipoylated enzymes induces their aggregation and subsequent loss of Fe-S cluster proteins, which triggers proteotoxic stress and ultimately leads to cell death. Whether the rapid cell death we observe in this study is fully due to cuproptosis can be debated. The concentration range that was used exceeds the concentration at which cuproptosis is observed in previous reports, and we did observe caspase activation *in vitro* and *in vivo*, suggesting downstream induction of apoptosis.

Previous research showed that cells with high mitochondrial respiration were more sensitive to ES treatment than cells that rely on anaerobic glycolysis.^39^ This is in apparent contrast with our results, in which we observed that the ES-sensitive CMS4 cells have lower mitochondrial respiration and fewer mitochondria compared to the ES-insensitive group. A possible explanation is that CMS4 lines lost redundancy in mitochondrial content during the epithelial-to-mesenchymal transition while being highly reliant on the few mitochondria that were left. Increased mitochondrial content in an isogenic setting indeed conferred to ES resistance, which bolsters this hypothesis.

Our study provides a class of compounds suitable for future intraperitoneal treatment in a patient population that currently faces a dismal prognosis. We propose that the repurposing of Cu ionophores could lead to improved efficacy of HIPEC in CRC-PMD and possibly for PMD of other cancer types as well.

### Limitations of the study

While we envision a clinical application in which ES is administered after CRS and in combination with the standard of care HIPEC, the *in vivo* experiments in this study did not capture the full scope of the clinical complexity of such intervention. The mouse experiments were conducted using immunodeficient mice, and ES-Cu was administered as a single intraperitoneal injection rather than as a true HIPEC procedure. In addition, promising but limited efficacy was observed in this model. To complement the mouse experiments, we applied the compound in an immunocompetent rat HIPEC model, in which the HIPEC procedure can be more accurately mimicked. However, no CRS was performed, and the completeness of CRS is a major indicator for progression-free survival. In the experimental setting, this is a possible confounder that is hard to control. Without CRS, complete eradication is hard to achieve, and this has yet to be seen. In addition, long-term monitoring of the rats following treatment will be essential to ascertain its true therapeutic potential and safety profile. Nevertheless, we showed that molecular markers of cell death are increased and markers of tumor growth reduced following application of ES-Cu. Prior to moving forward to early-phase clinical trials in resectable patients, future preclinical work should provide additional evidence for efficacy in the relevant treatment setting, such as a CRS-HIPEC model.

## STAR★Methods

### Key resources table

**Table:** 

REAGENT or RESOURCE	SOURCE	IDENTIFIER
**Antibodies**		

Cleaved Caspase 3 (Asp175) Antibody	Cell Signaling Technology	Cat# 9661S; RRID: AB_2341188
Recombinant Anti-Ki67 antibody	Abcam	Cat# ab16667; RRID: AB_302459
Poly-HRP-*anti*-Rabbit IgG	Immunologic	Cat# DPVR-55HRP; RRID:AB_2915958
DLAT	Cell Signaling Technology	Cat# 12362S; RRID: AB_2797893

**Bacterial and virus strains**		

pMD2.G	AddGene	12259
pMDLg/pRRE	AddGene	12251
pRSV-Rev	AddGene	12253
pLV-mitoDsRed	Addgene	44386

**Biological samples**		

FFPE embedded short- long-survivor samples	AMC	N/A

**Chemicals, peptides, and recombinant proteins**		

Matrigel	Corning	356213
RPMI	Gibco	52400–025
Advanced DMEM F/12	Gibco	12634010
IMDM	Gibco	12440053
Pen/Strep	Lonza/Gibco	DE17-602E/15070063
Primocin	Invitrogen	Ant-pm-1
Antibiotic-Antimycotic	Gibco	15240062
Glutamax	Gibco	35050–038
HEPES	Gibco/Lonza	15630-056/BE17-737E
β-mercaptoethanol	Sigma-Aldrich	CAS 60-24-2
Trace elements B	Corning	25-022-CI
Trace elements C	Corning	25-023-CI
Heparin	Sigma-Aldrich	H4784-250MG
Insulin	Sigma-Aldrich	I9278
Human bFGF	Peprotech	100-18B 1000
Human FGF10	Peprotech	100–26
Human EGF	Peprotech/Sigma-Aldrich	AF-100-15 1mg/E9644
N2 Supplement	Gibco	17502–048
B27 Supplement	Gibco	17504–044
MurineEGF	TEBU-BIO	315-09-B
R-spondin-1 conditioned medium	Prepared in-house	
Noggin-conditioned medium	Prepared in-house	
A83-01	Biovision/Bio-Techne (R&D Systems)	1725-1/2939
SB202190	Sigma-Aldrich	S7067
Y27632	Abmole bioscience	#27632
Nicotinamide	Sigma-Aldrich	N0636-100gr
Forskolin	Bio-Techne (R&D Systems)	1099
Gastrin	Sigma-Aldrich	G9145–0.5 mg
Prostaglandin E2	Tocris	2296–10
Heregulinβ-1	Peprotech	100–03
Hydrocortisone	Sigma-Aldrich	H0888
β-Estradiol	Sigma-Aldrich	E2257
Elesclomol	MedChemExpress	HY-12040
Copper(II)chloride	Sigma-Aldrich	222011-50G
Bathocuproinedisulfonic acid disodium salt	Sigma-Aldrich	146625
Disulfiram	MedChemExpress	HY-B0240
PDTC	Sigma-Aldrich	P-8765
MitoTracker Deep Red FM	ThermoFisher	M22426
MitoQ	Medkoo Biosciences	317102
N-acetylcysteine (NAC)	Sigma-Aldrich	A9165-25g
ESRRA inhibitor	Lead Pharma	LP 0821^28^
Lipofectamine 2000	Invitrogen	11668–019
MitoSOX Red mitochondrial superoxide indicator	Invitrogen	M36008/11579096
PowerDAB	Immunologic	BS04-999
Hematoxylin and eosin	Klinipath	4085–9002
Oligomycin	Sigma-Aldrich	O04876
FCCP	Sigma-Aldrich	C2920
Rotenone	Sigma-Aldrich	R8875
Antimycin A	Sigma-Aldrich	A8674
Oxaliplatin	Sigma-Aldrich	O9512
Mitomycin C	MedChemExpress	HY-13316
Cisplatin	Selleckchem	S1166
D-glucose	Sigma-Aldrich	G8769
Glucose		CAS 50-99-7
Galactose		CAS 59-23-4
DCFDA	Sigma-Aldrich	D6883-50MG
Hoechst	ThermoFisher	62249

**Critical commercial assays**		

AllPrep DNA/RNA/miRNA Universal Kit	Qiagen	80224
AllPrep DNA/RNA FFPE Kit	Qiagen	80234
CellTiter-Blue Cell Viability Assay	Promega	G8082
CellTiter-Glo 3D Luminescent Cell Viability Assay	Promega	G9682
JC-10	Abcam	Ab112134
CaspGLOW Fluorescein Active Caspase-3 Staining Kit	Biovision	K193-100
QuickExtract	Lucigen	QE09050
LightCycler® 480 SYBR Green I Master	Roche	4887352001
SMARTer® Stranded Total RNA-Seq Kit v3	Takara Bio	634485

**Deposited data**		

RNA-Seq data of CRC-PM patients	https://www.ncbi.nlm.nih.gov/geo/query/acc.cgi	GEO: GSE183202
RNA-Seq data short- and long-survivors CRC-PM	https://www.ncbi.nlm.nih.gov/geo/query/acc.cgi	GEO: GSE242676
Genomics of Drug Sensitivity in Cancer		Garnett et al.^18^

**Experimental models: Cell lines**		

HEK293T	ATCC	CVCL_0063
HUTU-80	ATCC	CVCL_1301
MDST8	ATCC	CVCL_2588
COLO-320HSR	ATCC	CVCL_0220
CaR-1	ATCC	CVCL_1116
OUMS-23	ATCC	CVCL_3088
HT55	ATCC	CVCL_1294
SW948	ATCC	CVCL_0632
RCM-1	ATCC	CVCL_1648
LS-1034	ATCC	CVCL_1382
LS411N	ATCC	CVCL_1385
LS513	ATCC	CVCL_1386
SW48	ATCC	CVCL_1724
SW620	ATCC	CVCL_0547
HCT116	ATCC	CVCL_0291
SNU-C1	ATCC	CVCL_1708
KM12	ATCC	CVCL_1331
NCI-H716	ATCC	CVCL_1581
T84	ATCC	CVCL_0555
LS180	ATCC	CVCL_0397
Hs766T	ATCC	CVCL_0334
PSN-1	Gift Prof. Peblani	CVCL_1644
PANC-1	ATCC	CVCL_0480
Panc89	Gift Prof. Knippschild	CVCL_4056
BxPC3	ATCC	CVCL_0186
AsPC-1	ATCC	CVCL_0152
Capan-1	ATCC	CVCL_0237
Capan-2	ATCC	CVCL_0026
HPAF-II	ATCC	CVCL_0313
TOR14	UMC Utrecht	*N/A*
TOR17	UMC Utrecht	*N/A*
PM03-1	UMC Utrecht	*N/A*
PM14-2	UMC Utrecht	*N/A*
TOR22	UMC Utrecht	*N/A*
PM02-1	UMC Utrecht	*N/A*
CC531	Gift Prof. de Hingh	CVCL_0206
*Lgr5-EGFP-IRES-Cre*^*ERT2*^ organoids (WT)	Sato et al.^33^	*N/A*
*villin*Cre^ER^ Kras^G12D/+^ Trp53 ^fl/fl^ Rosa26^N1icd/+^ tumoroids (KPN)	Jackstadt et al.^34^	*N/A*

**Experimental models: Organisms/strains**		

Athymic Nude-Foxn1^nu^ mice	Envigo RMS B.V.	Cat# 069; RRID: IMSR_ENV:HSD-069
WAG/Rij rats	Charles River	638

**Oligonucleotides**		

Fw *mTL1* cacccaagaacagggtttgt (mtDNA)	Sigma-Aldrich	*N/A*
Rv *mTL1* tggccatgggtatgttgtta (mtDNA)	Sigma-Aldrich	*N/A*
Fw *B2M* tgctgtctccatgtttgatgtatct (gDNA)	Sigma-Aldrich	*N/A*
Rv *B2M* tctctgctccccacctctaagt (gDNA)	Sigma-Aldrich	*N/A*

**Software and algorithms**		

Prism 9.5.1	Graphpad	www.graphpad.com
IncuCyte base analysis software	Sartorius	*N/A*
R2 bioinformatics platform	AMC	http://hgserver1.amc.nl
RSEM	Li et al.^40^	N/A
STAR aligner	Dobin et al.^41^	N/A
DESeq2	Love et al.^42^	N/A
CMS classifier	Guinney et al.^9^	https://github.com/Sage-Bionetworks/CMSclassifier
fGSEA	fGSEA^43^	https://bioconductor.org/packages/release/bioc/html/fgsea.html
QuPath version 0.2.3	QuPath	https://qupath.readthedocs.io/
FlowJo v10	Tree Star	*N/A*

**Other**		

Seahorse XF96 Extracellular Flux Analyzer	Agilent	Seahorse XFe96 Analyzer
Transmission electron microscope	ThermoFisher	FEI Tecnai T12
Confocal microscope	Leica	SP8-X
CytoFLEX-S FACS	BeckmanCoulter	B75442
FACS cell sorter	Sony	SH800
IncuCyte™ S3	Sartorius	4647
Plate spectrophotometer	BioTek Instruments	Biotek HT
EVOS FL Auto	ThermoFisher	AMAFD1000
LC480 II Lightcycler	Roche	05015243001
HP D300 Digital Dispenser	Tecan	N/A
2100 Bioanalyzer	Agilent Technologies	G2939BA
QIAcube	Qiagen	9001293
Qubit Fluorometer	ThermoFisher	Q33238
4200 TapeStation System	Agilent Technologies	G2991BA

### Resource availability

#### Lead contact

Further information and requests for resources and reagents should be directed to and will be fulfilled by the lead contact, Maarten Bijlsma (m.f.bijlsma@amsterdamumc.nl).

#### Materials availability

All unique/stable reagents generated in this study are available from the lead contact with a completed materials transfer agreement.

#### Data and code availability

RNA-Seq data have been deposited at Gene Expression Omnibus (GEO) and are publicly available as of the date of publication. Accession numbers are listed in the key resources table. No new software or custom code was developed as part of the research presented in this paper. The analyses were conducted using existing, publicly available software and tools, which are cited appropriately in the key resources table. For reproducibility and transparency, all the R scripts used in this study are available upon request and without restriction to the lead contact. Any additional information required to reanalyze the data reported in this paper is available from the lead contact upon request.

### Experimental models and study participant details

#### Patient samples

In this study we used clinical and RNAseq data from 82 previously published PM samples from 52 patients, collected at the Amsterdam University Medical Center, location VUmc between 2010 and 2018 (Amsterdam UMC cohort, GSE183202).^7^ Eligibility criteria for inclusion were histologically proven CRC with synchronous or metachronous PM, age older than 18 years and fresh frozen tissue available. Baseline characteristics are stated in Table S1. Additionally, we used clinical and RNAseq data from 28 patients with CRC-PM treated with CRS-HIPEC at the Catharina Hospital Eindhoven between 2012 and 2017 (CZE cohort, GSE242676). Eligibility criteria for inclusion were histologically proven CRC with synchronous or metachronous PM, age older than 18 years, PM FFPE material available and OS < 24 months or >36 months. Baseline characteristics are stated in Table S2. After completion of CRS, resection outcome was determined according to the maximal size of residual tumor tissue: an R1 (complete) resection was scored when no macroscopically visible tumor was left behind; an R2a resection was scored when the tumor was smaller than 2.5 mm; and an R2b resection was scored when the residual tumor was larger than 2.5 mm.^13^ All patient samples were collected according to Dutch research guidelines of the Federation of Dutch Medical Scientific Societies (FDMSS), as described in “Human Tissue and Medical Research: Code of Conduct for Responsible use”. When required, patients provided informed consent for sampling additional tumor tissue for study purposes.

#### Cell culture

CRC cell lines CaR-1, HT55, HUTU-80, LS180, OUMS-23, SW48, SW620, SW948 and T84 were cultured in DMEM/F-12 medium with L-glutamine, 15 mM HEPES (Thermo Fisher Scientific) supplemented with 10% v/v fetal bovine serum (Gibco), and penicillin-streptomycin (Lonza). Cell lines COLO-320HSR, HCT116, KM12, LS1034, LS411N, LS513, MDST8, NCI-H716, RCM-1 and SNU-C1 were cultured in RPMI 1640 with L-glutamine, 25 mM HEPES (Thermo Fisher Scientific) supplemented with 10% v/v fetal bovine serum (Gibco), penicillin and streptomycin, 1% D-glucose solution plus (Sigma-Aldrich) and 100 μM sodium pyruvate (Thermo Fisher Scientific). These cell lines were obtained from the Sanger Institute (Cambridge, UK).

Primary CRC cell lines Co108, DA13, GTG7 and RC511 were derived from colon cancer patients as described previously^44^ and cultured in advanced DMEM/F-12 (Thermo Fisher Scientific), supplemented with N2 supplement (Invitrogen, Waltham, MA, USA), 2 mM L-glutamine, 0.15% D-glucose (Sigma-Aldrich), 100 μM β-mercaptoethanol (Sigma-Aldrich), trace elements B and C (Thermo Fisher Scientific), 5 mM HEPES (Gibco), 2 μg/mL heparin (Sigma-Aldrich), 10 μg/mL insulin (Sigma-Aldrich), 10 ng/mL human bFGF and 20 ng/mL human EGF (Peprotech) in ultra-low attachment flasks (Corning).

CC531 cell line (kind gift from Prof. Ignace de Hingh, Catharina Cancer Institute (Eindhoven) and Roger Lomme, Radboud University (Nijmegen)) was cultured in RPMI1640 medium (Gibco) containing 25 mM HEPES and supplemented with 10% fetal bovine serum (Gibco) and 1% penicillin/streptomycin/glutamine (Gibco). PDAC cell lines Hs766T (ATCC), PSN-1 (kind gift from Prof. Peblani, University of Padova, Italy), PANC-1 (ATCC) were cultured in DMEM. Panc89 (kind gift from Prof. Knippschild, University hospital Ulm, Germany), BxPC3 (ATCC), AsPC-1 (ATCC), were cultured in RPMI. Capan-1 (ATCC), Capan-2 (ATCC) and HPAF-II (ATCC) were cultured in IMDM. All media were supplemented with 10% v/v fetal bovine serum (Gibco), penicillin and streptomycin and L-glutamine (2 mM).

All cell lines were monthly tested for mycoplasma. All human cell lines were authenticated by STR profiling. Cultures were normoxic at all times.

#### Organoids

CRC patient-derived organoids were generated from ascites fluid collected in the CRC-PIPAC trial (NCT03246321),^45^ this study was approved by the Medical Research ethics Committees United (MEC-U) in Nieuwegein, the Netherlands.^8^^,^^30^ The collection and processing of ovarian cancer patient-derived organoids was approved by the medical ethical committee UMC Utrecht (METC UMCU), in the biobanking protocol: 14–472 HUB-OVI.^46^ All PM-derived CRC organoids were cultured with CRC organoid growth medium and Matrigel (Corning). The CRC organoid growth medium consisted of Advanced DMEM/F12 (Gibco), supplemented with 400 μM Glutamax (Gibco), 10 mM HEPES (Lonza), and penicillin-streptomycin (Gibco),100 ng/mL Noggin conditioned medium, R-Spondin conditioned medium, B27 (Gibco), 500 nM A83-01 (Biovision), 10 μM SB202190 (Sigma-Aldrich), 1.25 mM N-acetyl-L-cysteine (Sigma-Aldrich), primocin, 10 mM Nicotinamide (Sigma-Aldrich), 50 ng/mL EGF (Sigma-Aldrich), 10 nM Gastrin [Leu15] (Sigma-Aldrich), and 10 nM prostaglandin E2 (Tocris). PM-derived ovarian cancer organoids were cultured in Advanced DMEM/F12 (Gibco), Noggin conditioned medium, R-Spondin conditioned medium, B27 (Invitrogen), 1.25 mM N-acetyl-L-cysteine (Sigma-Aldrich), 1:500 primocin (Invitrogen), 10mM Nicotinamide (Sigma-Aldrich), 500nM A83-01 (Bio-techne (R&D Systems)), 10 ng/mL FGF10 (Peprotech), 37.5 ng/mL Heregulinβ-1 (Peprotech), 5μM Y27632 (Abmole bioscience), 50 ng/mL hEGF (Peprotech), 10μM Forskolin (Bio-techne (R&D Systems)), 500 ng/mL Hydrocortisone (Sigma-Aldrich), 100nM β-estradiol (Sigma-Aldrich).

Murine organoids were established in^33^ and.^34^ We used the following medium formulation for WT organoids and KPN tumoroids to warrant equal culture conditions: Murine organoid medium consisting of advanced DMEM/F12 medium (Gibco) containing N2 (Gibco) and B27 (Gibco) supplements, Glutamax (Gibco), 5mM HEPES (Gibco), 1 mM N-acetyl-L-cysteine (Sigma-Aldrich) and antibiotic/antimycotic (ThermoFisher). The basal organoid medium was freshly supplemented with the following growth factors: mouse EGF 50 ng/mL (TEBU-BIO), R-spondin (conditioned medium) and Noggin (conditioned medium). Culture medium was refreshed twice a week.

#### Animal studies

All animal experiments were approved by the Animal Experimentation Committee at the Amsterdam UMC in Amsterdam, ethical approval numbers AVD11800202013801 (mice) and AVD1180020174184 (rats), and performed according to national guidelines. All animals were housed in a 12-h light/12-h dark cycle, with temperatures between 20°C and 24°C and 40–70% humidity.

For the mouse experiments, female nude (Hsd:Athymic Nude-Fox1^nu^) mice (6–12 weeks old) were purchased from Envigo. HUTU-80 and MDST8 cells (10.000 cells/mouse) in medium containing 50% Matrigel (Corning) were injected intraperitoneally. Two weeks after tumor cell injection, mice were randomized into experimental groups. Six weeks after tumor cell injection, mice were sacrificed followed by dissection including assessment of PM.

For the rat experiments, female WAG/Rij rats were purchased from Charles River Laboratories (8–10 weeks old). CC531 cells (2 × 10^6^ cells/rat) in medium were injected intraperitoneally. After injection, ultrasound was applied weekly to assess the tumor outgrowth in the rats. When most tumors reached a size of 4-6 mm, rats were randomized into experimental groups and HIPEC was performed.

### Method details

#### *In vivo* ES-Cu treatment

In mice, 2 weeks after tumor cell injection 1 mL of vehicle control or 25 μM ES-Cu solution was once injected intraperitoneally. Rats in the HIPEC arm were treated with 1.3 mg/kg ES-Cu in 500 mL physioneal 40 glucose 1.36% carrier solution. The perfusate solution was prepared and pre-heated using a thermostatically controlled water bath. Inflow temperatures of 38°C or 42°C were set to achieve peritoneal temperatures of 37°C or 41°C, respectively. To allow access to the peritoneal cavity, a small incision was made and the abdominal wall was attached to a plastic retractor ring with sutures. A semi-open in/outflow HIPEC construction was ensured by placing four perforated inflow tubes and one outflow catheter through the retractor. To start the HIPEC, the roller pump was set on 220 RPM, corresponding to 1.6 mL/s. After approximately 5–10 min the desired temperature was reached in all four quadrants. Subsequently, HIPEC was applied for 30 min. At the end of the HIPEC procedure, the in/outflow construction and retractor ring were removed and the abdominal wall and skin were closed with sutures. Rats received carprofen rimadyl before and after HIPEC to reduce pain and inflammation. The control rats remained untreated: they did not receive any form of infusion. This control mimics the clinical situation in which heated flushing without cytotoxics is never given, as well as previous preclinical work using comparable rat models.^36^

#### Cytotoxicity assays

Cells were seeded in a 96-well plate in triplicate in 100 μL of medium. After overnight attachment, drugs were added to the medium using a Tecan D300e Digital Dispenser. In case of continuous exposure experiments, plates were incubated with the drugs for 72 h at 37°C. In case of wash-out experiments, plates were incubated for indicated times with drug-containing medium at 37°C. After drug wash-out, cells were allowed to grow for 48 or 72 h.

For readout using CellTiter-Blue, 20 μL of CellTiter-Blue (Promega) was added. After 3 h of incubation, protected from light at 37°C, fluorescence signal was measured by a fluorescence reader (Biotek). For readout using IncuCyte microscopy, plates were placed in the IncuCyte S3 live cell analysis system (10× objective) after treatment and photomicrographs were taken at indicated time points. For the PDAC ES treatment experiment, cells were treated for 7 days. Cell numbers were analyzed by trypsinization and FACS based counting with CountBright Absolute beads (Thermo Fisher).

For organoid drug testing experiments, organoids were dissociated into single cells using TrypLE (Gibco, Thermo Fisher Scientific) and mechanical disruption. Single live cells (3000 per well) were seeded in a 1:1 mixture of growth medium and Matrigel (for CRC organoids TOR14, TOR17, TOR22 and PM02-1) or basement membrane extract (BME) (for CRC organoids PM08–1, PM14-2 and ovarian organoids), and allowed to form organoids for 3 days. The indicated drugs were then added at indicated concentrations using a Tecan D300e Digital Dispenser. For CRC organoids, all conditions were plated in quadruplo. For ovarian organoids, all conditions were plated in triplo. The plates were incubated for 30 min or 60 min at 37°C. After drug wash-out, organoids were allowed to grow for 6 days and cell viability was assessed by CellTiter-Glo on a SpectraMax Microplate Reader.

#### ICP-MS

For the detection of Cu, ICP-MS was conducted by Medlon B.V. Sample preparation was performed as follows: For *in vitro* cultures, 2 × 10^6^ cells were lysed using 1000 μL RIPA buffer (product). 25 μL was used to quantify protein input using a BCA kit (prod). For mouse experiments, tumor tissue samples were collected 3 h after ES-Cu treatment and snap-frozen until further processing. For rats, animals were sacrificed and tumors were collected directly after HIPEC for analysis of Cu levels. At the time of analysis, samples were weighed to quantify the input. To digest the tissue sample, 20 μL of 2% nitric acid was added to the tube and incubated at 95°C for 10 min. After digestion, the samples were diluted 1:50 with 2% nitric acid.

#### Flow cytometry

To assess caspase activation by flow cytometry (FACS), cells were treated with 2.5 μM ES (1:2 CuCl_2_) for the indicated time points and then harvested with trypsin-EDTA (Lonza, Basel, Switzerland). CaspGLOW Fluorescein Active Caspase-3 Staining Kit (Biovision, K193-100) was diluted 1:300 CaspGLOW in regular cell culture medium. The cells were stained for 1 h at 37°C. For flow cytometric detection of mitochondrial ROS production, cells were stained prior to treatment with 5 μM MitoSOX (Invitrogen, M36008/11579096) in regular cell culture medium for 10 min at 37°C. Subsequently, cells were washed and treated with vehicle control or 2.5 μM ES (1:2 CuCl_2_). Cells were washed with FACS buffer (2% FCS in PBS) prior to flow cytometry analysis. Data were analyzed using FlowJo 10.

#### Confocal microscopy

Cells were seeded on coverslips in flat-bottom 6-well plates and allowed to attach overnight. Cells were then exposed to 30 min of ES-Cu (500 nM). 24 h later, cells were washed with PBS, fixed with 2% paraformaldehyde (Thermo Fisher) in PBS, and permeabilized with PBS containing 0.1% Triton X-100 (Merck) and 1% FBS. Immunostaining of DLAT was performed in permeabilization buffer by a 1:100 incubation with mouse anti-DLAT (Cell Signaling) for 1 h, washing with PBS, and a 30-min 1:150 incubation with anti-mouse Cy3 (Jackson). Samples were counterstained by incubation with Hoechst 33342 at 5 μg/mL for 10 min, and mounted on glass slides using Vectashield (Vectorlabs). Z-stacks were captured using a Leica DM6i wide-field fluorescence microscope equipped with appropriate filtersets, a 40x/1.25/0.75 Plan Achromat oil objective (Leica) and a Leica DFC9000 GT camera.

#### Mitochondrial depolarization measurement

20,000 HUTU-80 cells were seeded in a 96-well plate in triplicate in 100 μL medium. After overnight attachment, cells were treated with ES (2.5 μM) combined with CuCl_2_ (5 μM) 8, 5, 2, 0 h before the addition of JC-10 (Abcam, #ab112134). Trifluoromethoxy carbonylcyanide phenylhydrazone (FCCP) (1 μM) was added 15 min before the addition of JC-10, and functions as positive control. After 30 min of incubation at 37°C and protected from light, fluorescence signal was measured by a fluorescence reader (Biotek): excitation = 540 nm, emission = 590 nm for aggregated imaging, and excitation = 490 nm, emission = 525 nm for monomeric imaging. The mitochondrial depolarization was measured by the ratio of green monomeric to red aggregated fluorescence.

#### Seahorse XF96 Extracellular Flux Analyzer

The Seahorse XF96 Extracellular Flux Analyzer (Seahorse Biosciences) was used to obtain real-time measurements of oxygen consumption rate (OCR) and extracellular acidification rate (ECAR) in cells. Cells were seeded in 96-wells Seahorse culture plates at a density between 25,000 and 50,000 cells/well and were reconstituted in culture medium overnight. Prior to the analysis, the culture medium was replaced with an Assay Medium (DMEM, 15 mM glucose, sodium pyruvate (0.5 mM) and L-glutamine (2 mM). Supplemented assay medium was adjusted to pH 7.4 and maintained at 37°C throughout the experiment. After baseline metabolic flux readings were established through 3 consecutive baseline measurements, ES:CuCl_2_ (5 μM:5 μM) or control (5 μM CuCl_2_, 5 μM ES, or dH_2_O) were injected. Subsequent changes to OCR and ECAR levels were assessed over minimally 3 further measurements. For the “Mito Stress Test” protocol oligomycin, FCCP, and antimycin A & rotenone were sequentially injected according to the manufacturer’s instructions.

#### Quantification of mitochondrial copy number

Mitochondrial DNA copy number was determined by qPCR of total extracted DNA by measuring the ratio of mitochondrial gene mt-TL1 (Fw: 5′ CACCCAAGAACAGGGTTTGT 3′, Rv: 5′ TGGCCATGGGTATGTTGTTA 3′) and nuclear gene B2M (Fw: 5′ TGCTGTCTCCATGTTTGATGTATCT 3′, Rv: 5′ TCTCTGCTCCCCACCTCTAAGT 3′). Total DNA was obtained by QuickExtract Extraction Solution (Lucigen, Middleton, USA) according to the manufacturer’s protocol. The qPCR reaction was performed with SYBR Green (Roche Life Science) using the Lightcycler 480 II (Roche Life Science).

#### Electron microscopy

Cells were treated at indicated time points with ES-Cu chloride or control. All samples were fixed in 0.1 M PHEM buffer, 2% paraformaldehyde (PFA), and 0.2% glutaraldehyde for minimally 4 h at room temperature, and subsequently washed and stored with PBS with 0.1 M PHEM buffer and 0.5% PFA at room temperature. For epon embedding, the cells were pelleted and dehydrated in an alcohol series and embedded into epon resin. With an ultramicrotome the cells were sectioned in 70–200 nm coupes and collected onto formvar coated 200 Mesh copper grids. The grids were contrasted for electron microscopy with uranyl acetate and counterstained with lead citrate. After staining the grids were imaged using a Thermo Fisher Tecnai 12 transmission electron microscope.

#### FACS sorting

Cells with different mitochondrial contents were sorted based on the labeling of pLV-mitoDsRed (Addgene) or MitoTracker Deep Red FM (Thermo Fisher Scientific).

Briefly, lentiviruses encoding pLV-mitoDsRed were produced in HEK293T cells with packaging vectors (pRSV-Rev, pVGSV and pMDLg/pRRE) using Lipofectamine 2000 (manufacturer). Lentiviruses particles were collected 24 h and 48 h post-transfection and used to infect CRC cells in the presence of 10 μg/mL polybrene. To obtain single-cell clones, single-cells were sorted in a 96-wells plate. Subsequently, the cells were expanded to and maintained in a T25 flask. MitoTracker Deep Red FM was added in the medium of 80% confluent CRC cells at the final concentration of 50 nM, and incubated for 20 min. By FACS sorting, the top 25% cell population and the bottom 25% cell population in terms of mitochondrial content were collected for cell viability assays.

#### Immunohistochemistry

Rats were sacrificed and tumors were collected 48 h post-HIPEC for assessment of apoptosis and cell proliferation. Proliferation and apoptosis in the tumor nodules were assessed by IHC stainings for Ki-67 and cleaved caspase-3, respectively. Sections of 4 μm were cut, deparaffinized and heat-induced antigen retrieval was performed with 1x Sodium Citrate pH 6 at 98°C, followed by peroxidase blocking. Slides were incubated with the primary antibody Recombinant Anti-Ki-67 antibody 1:200 (anti-rabbit, Abcam) or cleaved caspase-3 1:200 (anti-rabbit, Cell Signaling) overnight at room temperature. The next day, sections were incubated with the secondary antibody Poly-HRP-*anti*-Rabbit IgG (Immunologic) for 60 min at room temperature. Afterward, the sections were stained with PowerDAB (Immunologic), counterstained with hematoxylin (Klinipath) and mounted with Pertex. Representative pictures were taken with a light microscope (Olympus) at a magnification of 40×. Percentage positive staining was analyzed using the software program QuPath version 0.2.3.

#### RNA isolation of patient samples

For the Amsterdam UMC cohort, RNA was isolated and processed as described before.^7^ In brief, frozen tissue samples were cut in 20-μm-thick cryosections with a cryostat up to about 30 mg for each sample. Frozen samples were immersed in RLT buffer (AllPrep DNA/RNA/miRNA Universal Kit, Qiagen) and disrupted and homogenized using TissueLyser LT (Qiagen). Total RNA and DNA were isolated simultaneously from tissue lysates using the AllPrep DNA/RNA/miRNA Universal Kit (Qiagen), following the manufacturer’s instructions. The RNA integrity was measured using the Agilent RNA 6000 Nano Kit on an Agilent 2100 Bioanalyzer (Agilent Technologies). Only samples with an RIN (RNA integrity number) > 6.7 were subjected to further analysis. For the CZE cohort, total RNA was extracted from FFPE tumor samples using the AllPrep DNA/RNA FFPE kit (Qiagen), following the manufacturer’s protocols. The QIAcube classic system (Qiagen) was employed for automated sample processing. Nucleotide concentrations in the samples were quantified using the Qubit fluorometer (ThermoFisher), following the manufacturer’s guidelines. To assess RNA quality, the percentage of transcripts exceeding 200 base pairs in length (DV200) was determined using the Agilent Technologies 4200 TapeStation System (Agilent Technologies). Samples with a DV200 value greater than 15% were considered suitable for RNA sequencing.

### Quantification and statistical analysis

#### RNA-sequencing analysis

For the Amsterdam UMC cohort, RNA sample preparation and sequencing were performed as described before.^7^ In brief, libraries were prepared using Kapa mRNA HyperPrep, sequencing was performed using Illumina HiSeq (single read, 50 bp). RNA-seq transcript quantification was performed by mapping the high-quality reads to the GRCh38 human transcriptome by using the software RSEM^40^ and the STAR aligner.^41^ For the CZE cohort, library preparation and sequencing were outsourced to GenomeScan (Leiden, the Netherlands). RNA sample preparation was performed using the SMARTer Stranded Total RNA-Seq Kit v3 - Pico (Takara Bio). This kit is specifically designed for use with degraded and low-input RNA samples. Paired-end deep sequencing was conducted using the NovaSeq6000 sequencing platform, generating 100 million 150 base-pair reads per sample.

CMS classification of patient samples was performed based on gene profile expression as described before.^9^ For both cohorts, raw count matrix of RNA-seq was transformed by variance stabilizing transformation (DESeq2)^42^ and gene identifiers were converted from gene Symbol to Entrez gene ids using the R package org.Hs.e.g.,.db (genome-wide annotation for Human. R package version 3.8.2. Carlson, 2019). The processed matrix was used as input for the ‘single-sample predictor’ (SSP) classifier, part of the (‘CMSclassifier’ v1.0.0) R package, setting the option method = “SSP”. CMS classification of cell lines was used as described previously.^47^ For the geneset enrichment analysis, CRC-PM (GSE183202),^7^ primary CRC (GSE33113)^25^ and CRC cell line (E-MTAB-3610)^48^ data was used. All Hallmark gene sets^49^ were considered. Enrichment and statistical significance with Benjamini-Hochberg correction were calculated by the R package fgsea.^43^

#### Drug data analysis

Cell line sensitivity data provided by the Genomics of Drug Sensitivity data in Cancer (GDSC) project were downloaded (www.cancerrxgene.org/downloads, Release 7.0, Mar 2018).^50^ Cell line classification as previously described was used.^47^ Z-scores of the lC50 values of CMS2/3 vs. CMS4 classified cell lines were compared.

#### Statistical analysis

Statistical tests are explicitly stated in main text or figure legends. To summarize the statistical tests. We compared group means by unpaired two-tailed Student’s t test or one-way ANOVA. We compared survival distributions between two groups by a log rank test. A *p-*value of ≤0.05 was considered statistically significant.

## Supplemental information

Document S1. Figures S1‒S5 and Tables S1‒S4

Document S2. Article plus supplemental information
