# Supplementary material for: Exploiting a subtype-specific mitochondrial vulnerability for successful treatment of colorectal peritoneal metastases
Source: Cell Rep Med. 2024 Apr 25;5(5):101523. doi: 10.1016/j.xcrm.2024.101523 (PMC11148637; doi:10.1016/j.xcrm.2024.101523)
Supplement: Document S1. Figures S1‒S5 and Tables S1‒S4 [file mmc1.pdf]

Cell Reports Medicine, Volume *5*

Supplemental information

Exploiting a subtype-specific mitochondrial vulnerability for successful treatment of colorectal peritoneal metastases

Sanne Bootsma, Mark P.G. Dings, Job Kesselaar, Roxan F.C.P.A. Helderman, Kyah van Megesen, Alexander Constantinides, Leandro Ferreira Moreno, Ellen Stelloo, Enzo M. Scutigliani, Bella Bokan, Arezo Torang, Sander R. van Hooff, Danny A. Zwijnenburg, Valérie M. Wouters, Vincent C.J. van de Vlasakker, Laskarina J.K. Galanos, Lisanne E. Nijman, Adrian Logiantara, Veronique L. Veenstra, Sophie Schlingemann, Sterre van Piggelen, Nicole van der Wel, Przemek M. Krawczyk, Johannes J. Platteeuw, Jurriaan B. Tuynman, Ignace H. de Hingh, Jan P.G. Klomp, Arthur Oubrie, Petur Snaebjornsson, Jan Paul Medema, Arlene L. Oei, Onno Kranenburg, Clara C. Elbers, Kristiaan J. Lenos, Louis Vermeulen, and Maarten F. Bijlsma

SUPPLEMENTAL FIGURES

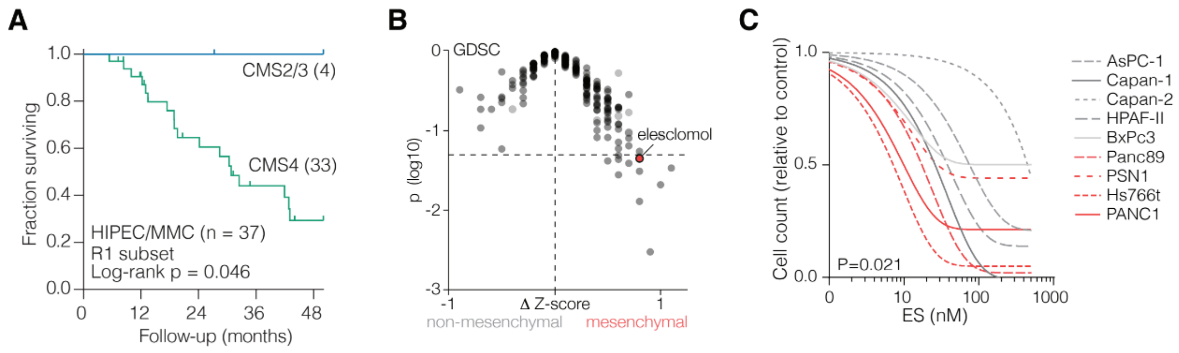

Figure S1 | Mesenchymal PDAC cell lines show increased sensitivity to ES treatment. Related to Figure 1.

A Kaplan-Meier overall survival analysis of patients treated with CRS-HIPEC with MMC and resection score R1, stratified by CMS2/3 (blue) and CMS4 (green) (log-rank test, p=0.046).

B Drug sensitivity of classified PDAC cell line data from GDSC. Shown on x-axis is the relative sensitivity; positive Z-score correlates with high relative sensitivity in mesenchymal cell lines. Y-axis indicates significance (P-value) by t-test of differential sensitivity. Bottom right hand corner includes drugs significantly more effective against mesenchymal cell lines.

C Indicated PDAC cell lines representing the mesenchymal and non-mesenchymal subtype were treated for 7 days with ES, after which cells were counted by bead-calibrated flow cytometry. Curves were fitted with non-linear regression, dose response curves. P-value (by ANOVA) is shown for pooled mesenchymal cell lines (n=4, shown in pink) versus pooled non-mesenchymal cell lines (n=5, shown in grey).

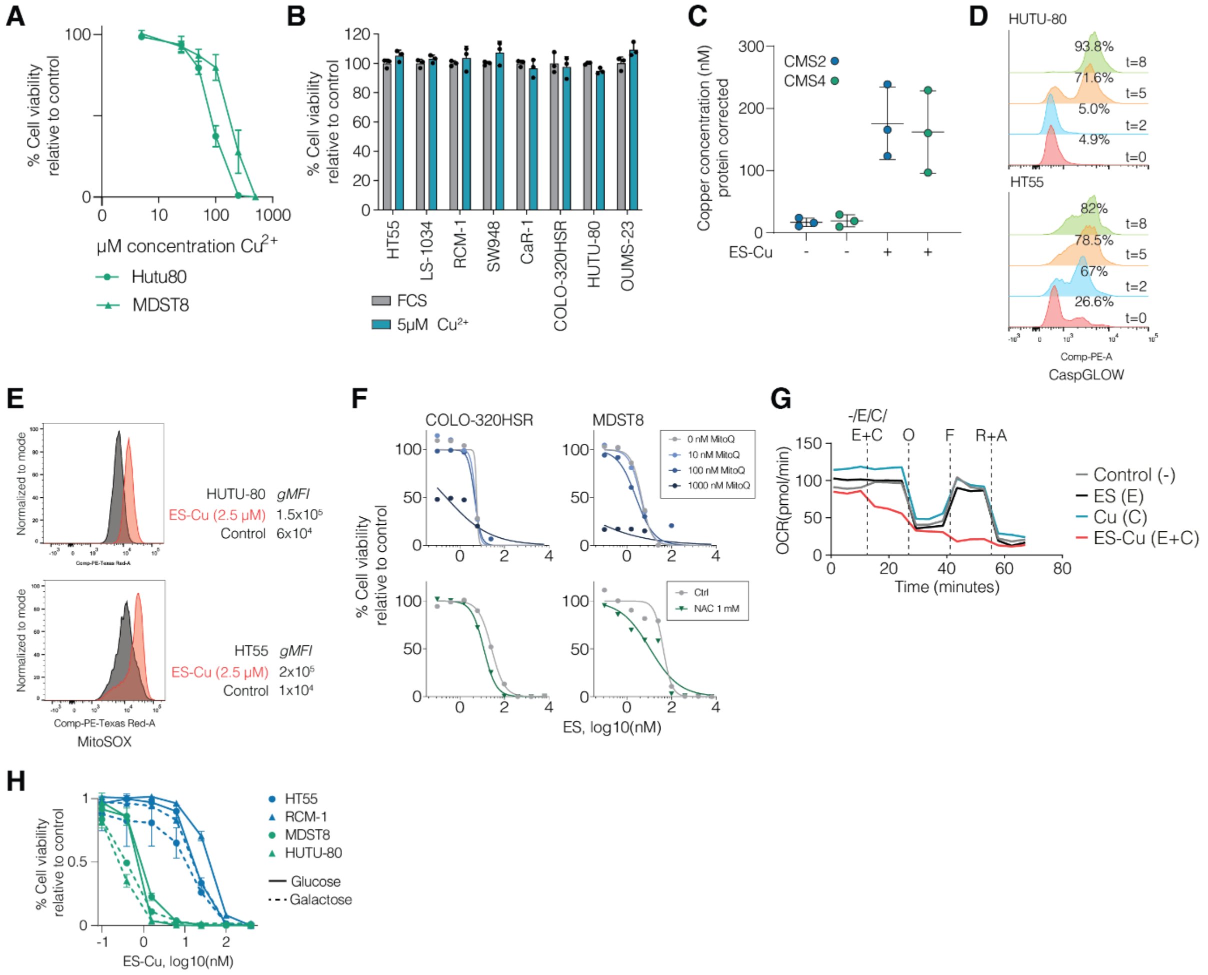

Figure S2 | Antioxidants do not prevent cell death following treatment with ES. Related to Figure 2.

A Dose-response curves of HUTU-80 and MDST8 cells treated with indicated doses of $CuCl_2$. Cell viability was assessed after 72 h by CellTiter-Blue. Data are means ± SD of 3 biological replicates, normalized to solvent control.

B Cell viability was assessed after cells were cultured with regular complete medium (control) or with 5 µM Cu^{2+} supplemented to the medium for 72 h.

C Cu levels after treatment with 2.5 µM ES-Cu or vehicle, determined by ICP-MS in CMS2 (n=3) and CMS4 (n=3) cell lines. Each dot represents one different cell line.

D Caspase-3/7-activation after treatment with 2.5 µM ES + 5 µM $CuCl_2$ at indicated time points (time in h) or vehicle in HUTU-80 and HT55 as determined by flow cytometry.

E Superoxide levels after 1 h treatment with 2.5 µM ES + 5 µM $CuCl_2$ or vehicle in HUTU-80 and HT55 as determined by flow cytometry.

F Cell viability from ES-Cu treated COLO-320HSR and MDST8 cells incubated with various antioxidants. The data represent the mean of three biological replicates.

G Seahorse flux analysis of HUTU-80 cells to measure the real-time oxygen consumption rate (OCR). Regular assay medium (control), 5 µM ES, $CuCl_2$ or the combination of Elesclomol and copper (ES-Cu) was added at t=12 min by injection in the analyzer. In the control no copper was present at all time. Regular mito stress test was applied: oligomycin to inhibit ATP synthesis, FCCP to uncouple the mitochondria, and rotenone/antimycin A to block the mitochondrial electron transport chain. Compounds were added at indicated times. O, oligomycin; F, FCCP; R+A, rotenone with antimycin A.

H Cell viability from ES-Cu treated CRC cell lines incubated in galactose or glucose supplemented medium. A fixed concentration of 5 µM $CuCl_2$ was supplemented to the culture medium. The data represent the mean ± SD from 3 biological replicates. Blue represents CMS2 subtype and green represents CMS4.

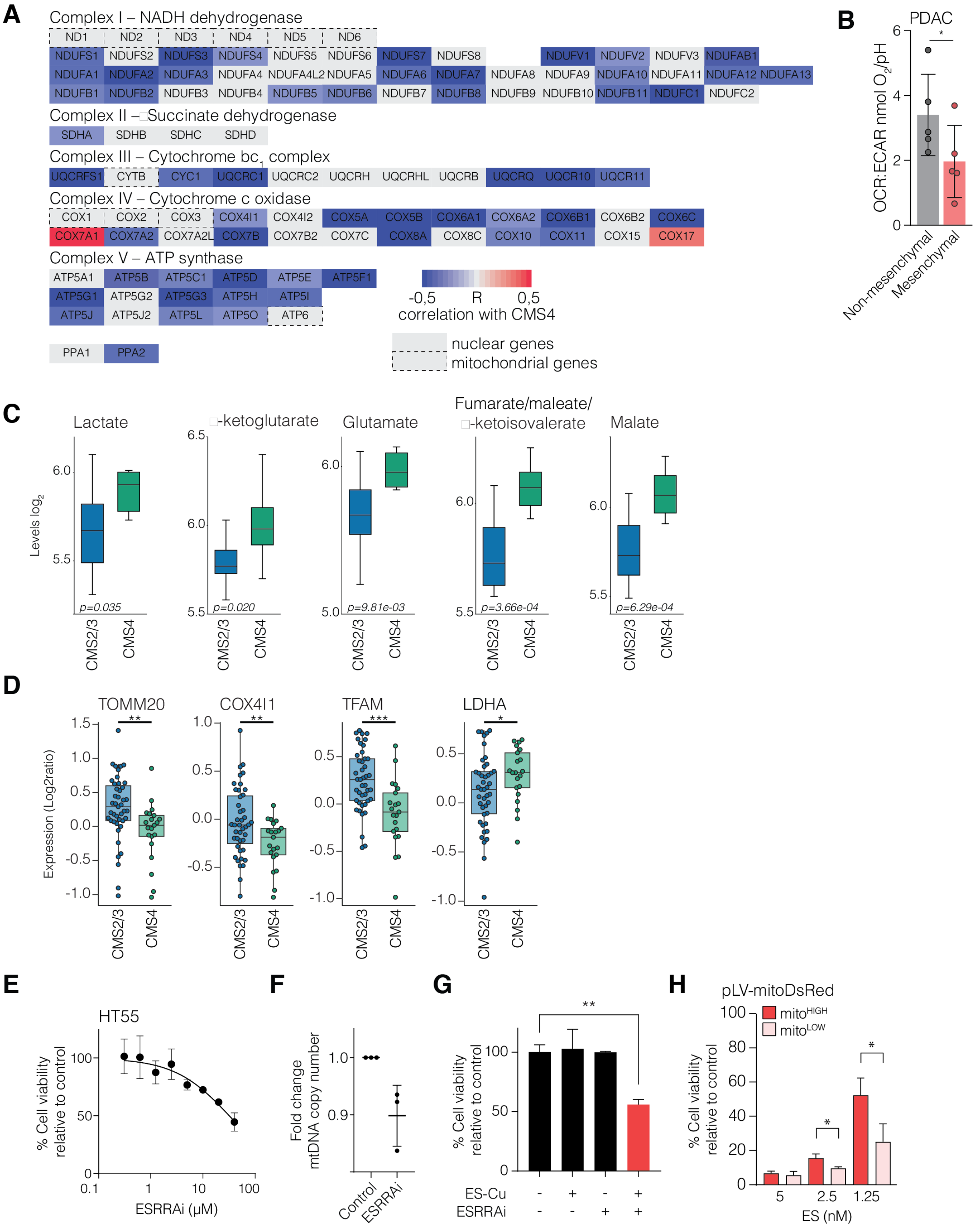

Figure S3 | Relationship between mitochondria, CMS4 and elesclomol sensitivity. Related to Figure 3.
A Heatmap representation of the relative expression of oxidative phosphorylation subunits in CMS4 CRC.
B Results from Seahorse analysis measuring the baseline ratio of OCR:ECAR in 10 PDAC cell lines, grouped by subtype. Each dot represents an independent cell line. Statistical analysis using unpaired t-test, $*p \leq 0.05$.
C Box plots depicting differentially detected metabolites in CMS2/3 vs CMS4 cell lines. Data represent the mean ± SD from CMS2/3 (n=14) vs CMS4 (n=6) cell lines, statistical analysis using unpaired t-test. Data from the CCLE metabolomics dataset[26].
D Box plots depicting differentially expressed proteins between CMS2/3 vs CMS4 patients. Data from the CPTAC proteomics dataset.[27] Protein expression (Log2ratio) was calculated as the ratio of sample abundance to reference abundance using the summed reporter ion intensities from peptides that could be uniquely mapped to a

gene. The relative abundances were then log2 transformed and zero-centered for each gene to obtain final, relative abundance values. For further details, we refer to the original paper.[27] Data represent the mean ± SD, statistical analysis using unpaired t-test.
E Dose-response curves of HT55 cell lines treated with ESRRA inhibitor for 72 h. Data are means ± SD of 3 biological replicates, normalized to solvent control.
F Relative mitochondrial abundance after 72 h treatment of control or ESRRAi (5 µM), quantified by mtDNA copy number. Data represents the means ± SD of 3 technical replicates.
G Cell viability of HT55 cells after 72 h treatment of control, ES-Cu (100 nM), ESRRAi (5 µM), and the combination of ES-Cu (100 nM) and ESRRAi (5 µM). Data are means ± SD of 3 biological replicates, normalized to control. Statistical analysis using unpaired t-test.
H Bar graphs depicting the cell viability 72 h after treatment with indicated concentrations of ES in sorted populations using HUTU-80 cells that were transduced with pLV-mitoDsRed (mito^{LOW} and mito^{HIGH}). Data are means ± SD of three biological replicates.

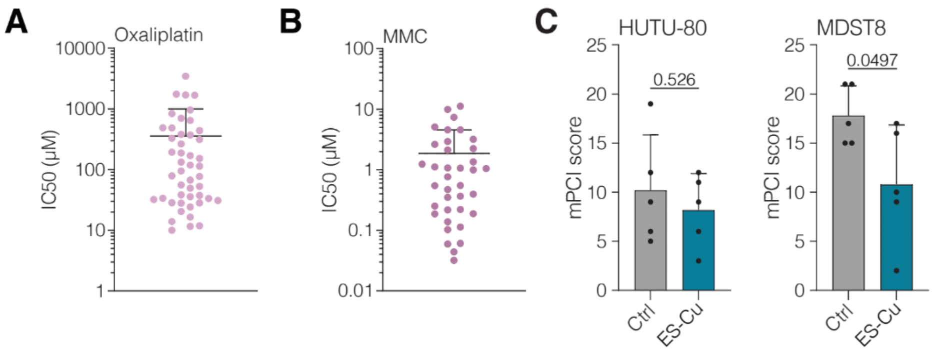

Figure S4 | In vivo treatment with ES-Cu. Related to Figure 4.
A IC50 values for Oxaliplatin of CRC cell lines in the GDSC dataset are presented. Each dot represents the IC50 for an individual CRC cell line (n=47). The large whisker represents the mean and the smaller whisker represents the upper SD.
B As for panel A, showing Mitomycin C IC50s (n=37).
C Immune deficient mice were grafted with HUTU-80 or MDST8 cells. After 2 weeks, mice received a single intraperitoneal 1 ml injection of vehicle control or 25 µM ES-Cu. After an additional 4 weeks, mice were sacrificed and tumor burden was assessed using the mPCI. Statistical analysis using unpaired t-test.

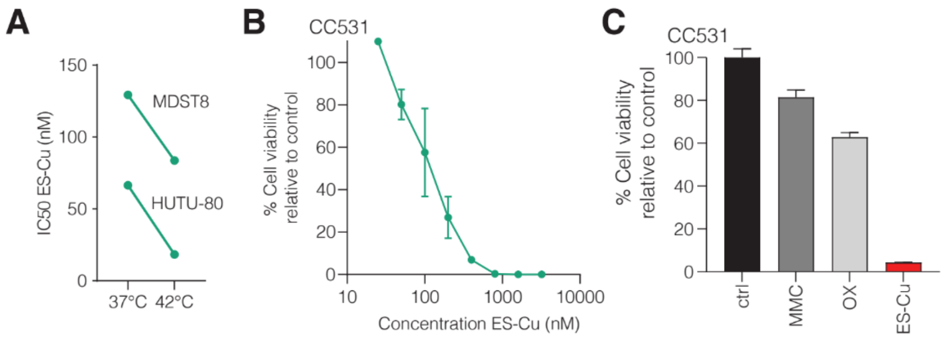

Figure S5 | Increased sensitivity of CMS4 cells to ES-Cu in hyperthermia. Related to Figure 5.
A IC50 of CMS4 cell lines HUTU-80 and MDST8 following 60 min ES-Cu treatment at 37°C or 42°C. After washing, cells were left to grow for 72 h after which cell viability was assessed by CellTiter-Blue.
B Dose-response curve of ES-Cu treated CC531 cells. Cells were treated for 60 min. After washing, cells were left to grow for 72 h after which cell viability was assessed by CellTiter-Blue.
C Cells were exposed to 60 min of ES-Cu (2.5 µM), MMC (2.5 µM) and OX (400 µM). After washing, cells were left to grow for 48 h after which cell viability was assessed by CellTiter-Blue. Data are means ± SD of minimally 3 biological replicates.

SUPPLEMENTAL TABLES

Table S1 | Baseline characteristics of HIPEC treated patients in Amsterdam UMC cohort. Related to Figure 1.
For categorical variables, numbers and proportions per subgroup were presented. Continuous variables were described by median and range. Continuous variables were compared using Mann-Whitney test. Categorical variables were tested using Chi-square test. R1 = no macroscopic residual tumor, R2a = macroscopic residual tumor smaller than 2.5 mm, R2b = macroscopic residual tumor larger than 2.5 mm

		**CMS2/3**	**CMS4**	**p-value**
No. of cases		4	35	
Gender	Male	1 (25%)	16 (45.7%)	0.429
	Female	3 (75%)	19 (54.3%)	
Age at HIPEC		57.5 (56-69)	65 (33-79)	0.327
ASA classification	I-II	3 (75%)	27 (77.1%)	0.923
	III	1 (25%)	8 (22.9%)	
PCI score		9.5 (6-16)	13 (2-22)	0.480
Resection score	R1	4 (100%)	33 (94.3%)	0.624[a]
	R2a		1 (2.9%)	
	R2b		1 (2.9%)	

[a] Result of R1 vs. R2 (R2a and R2b pooled)

Table S2 | Baseline characteristics of Eindhoven cohort. Related to Figure 1.
For categorical variables, numbers and proportions per subgroup were presented. Continuous variables were described by median and range. Continuous variables were compared using Mann-Whitney test. Categorical variables were tested using Chi-square test. R1 = no macroscopic residual tumor.

		**Short-term survivors**	**Long-term survivors**	**p-value**
No. of cases		15	13	
Gender	Male	8 (53.3%)	5 (38.5%)	0.431
	Female	7 (46.7%)	8 (61.5%)	
Age at HIPEC		68 (41-73)	69 (57-81)	0.277
ASA classification	I-II	13 (86.7%)	10 (78.6%)	0.502
	III	2 (13.3%)	3 (21.4%)	
PCI score		6 (1-18)	7 (1-21)	0.973
Resection score	R1	9 (60.0%)	9 (71.4%)	0.611
	Unknown	6 (40.0%)	4 (28.6%)	
CMS subtype	CMS2		2 (14.3%)	**0.049**[a]
	CMS3		1 (7.1%)	
	CMS4	15 (100%)	10 (78.6%)	

[a] Result of CMS2/3 vs. CMS4 (CMS2 and CMS3 pooled)

Table S3 | Geneset enrichment analysis results from tested genesets. Related to Figure 3.

GS DETAILS	SIZE	ES	NES	NOM p-val	FDR q-val	FWER p-val	RANK AT MAX
**HALLMARK_EPITHELIAL_MESENCHYMAL_TRANSITION**	**198**	**0.77**	**2.17**	**0**	**0**	**0**	**2923**
HALLMARK_TNFA_SIGNALING_VIA_NFKB	199	0.68	1.92	0	0	0	3701
HALLMARK_ALLOGRAFT_REJECTION	196	0.68	1.92	0	0	0	2609
HALLMARK_INFLAMMATORY_RESPONSE	200	0.65	1.84	0	0	0	3723
HALLMARK_ANGIOGENESIS	36	0.74	1.84	0	0	0	1748
HALLMARK_INTERFERON_GAMMA_RESPONSE	198	0.64	1.8	0	0	0	3982
HALLMARK_IL6_JAK_STAT3_SIGNALING	87	0.65	1.76	0	0	0	3122
HALLMARK_UV_RESPONSE_DN	141	0.63	1.76	0	0	0	3979
HALLMARK_COAGULATION	138	0.62	1.73	0	0	0.001	3680
HALLMARK_KRAS_SIGNALING_UP	199	0.6	1.71	0	0	0.001	3715
HALLMARK_MYOGENESIS	199	0.6	1.69	0	0	0.002	5250
HALLMARK_APICAL_JUNCTION	200	0.58	1.65	0	0	0.005	3133
HALLMARK_HYPOXIA	197	0.57	1.62	0	0.001	0.01	4421
HALLMARK_INTERFERON_ALPHA_RESPONSE	95	0.59	1.62	0	0.001	0.011	4272
HALLMARK_IL2_STAT5_SIGNALING	199	0.56	1.6	0	0.001	0.017	4619
HALLMARK_COMPLEMENT	200	0.56	1.59	0	0.001	0.02	4479
HALLMARK_APOPTOSIS	160	0.55	1.56	0	0.002	0.032	3473
HALLMARK_TGF_BETA_SIGNALING	54	0.58	1.52	0.005	0.003	0.068	6037
HALLMARK_HEDGEHOG_SIGNALING	36	0.57	1.43	0.029	0.011	0.232	2210
HALLMARK_APICAL_SURFACE	44	0.54	1.37	0.046	0.023	0.45	1656
HALLMARK_ESTROGEN_RESPONSE_EARLY	198	0.46	1.31	0.007	0.049	0.739	4865
HALLMARK_NOTCH_SIGNALING	32	0.54	1.31	0.097	0.051	0.769	4918
HALLMARK_P53_PATHWAY	196	0.43	1.23	0.047	0.132	0.98	5902
HALLMARK_ANDROGEN_RESPONSE	99	0.44	1.2	0.131	0.175	0.996	5474
HALLMARK_UV_RESPONSE_UP	156	0.42	1.19	0.105	0.182	0.997	2966
HALLMARK_KRAS_SIGNALING_DN	198	0.41	1.15	0.14	0.269	1	5085
HALLMARK_ESTROGEN_RESPONSE_LATE	198	0.4	1.13	0.175	0.312	1	4865
HALLMARK_ADIPOGENESIS	199	0.39	1.1	0.232	0.371	1	3488
HALLMARK_CHOLESTEROL_HOMEOSTASIS	73	0.4	1.07	0.374	0.462	1	6343
HALLMARK_XENOBIOTIC_METABOLISM	197	0.37	1.05	0.359	0.49	1	3245
HALLMARK_HEME_METABOLISM	192	0.37	1.04	0.381	0.498	1	6680
HALLMARK_REACTIVE_OXYGEN_SPECIES_PATHWAY	49	0.35	0.91	0.649	0.87	1	2071
HALLMARK_MITOTIC_SPINDLE	198	0.3	0.86	0.852	0.962	1	4659
HALLMARK_PROTEIN_SECRETION	95	0.3	0.83	0.824	0.982	1	5044
HALLMARK_GLYCOLYSIS	199	0.29	0.82	0.899	0.964	1	4987
HALLMARK_WNT_BETA_CATENIN_SIGNALING	42	0.31	0.78	0.829	0.988	1	6125
HALLMARK_BILE_ACID_METABOLISM	112	0.25	0.7	0.958	1	1	3979
HALLMARK_PI3K_AKT_MTOR_SIGNALING	104	0.25	0.69	0.963	0.995	1	4224
HALLMARK_UNFOLDED_PROTEIN_RESPONSE	110	0.24	0.67	0.973	0.977	1	5433
HALLMARK_PEROXISOME	104	-0.2	-0.86	0.902	0.818	0.64	4396
HALLMARK_FATTY_ACID_METABOLISM	156	-0.2	-0.96	0.579	0.616	0.501	2760
HALLMARK_MTORC1_SIGNALING	197	-0.2	-0.98	0.625	0.609	0.46	2214
HALLMARK_PANCREAS_BETA_CELLS	40	-0.3	-1.13	0.21	0.294	0.201	2204
HALLMARK_DNA_REPAIR	150	-0.3	-1.13	0.115	0.262	0.204	4730
HALLMARK_SPERMATOGENESIS	133	-0.3	-1.2	0.031	0.208	0.127	3616
HALLMARK_G2M_CHECKPOINT	194	-0.4	-1.85	0	0.002	0.001	3677
**HALLMARK_OXIDATIVE_PHOSPHORYLATION**	**200**	**-0.4**	**-1.86**	**0**	**0.002**	**0.001**	**5152**
HALLMARK_MYC_TARGETS_V2	58	-0.5	-1.99	0	0.001	0	4674
HALLMARK_E2F_TARGETS	198	-0.6	-2.48	0	0	0	4512
HALLMARK_MYC_TARGETS_V1	196	-0.6	-2.63	0	0	0	5867

Table S4 | Characteristics of PM organoids. Related to Figure 4.

organoid ID	location PM	obtained during	gender	age	TNM 1°CRC	location 1° CRC	histology
TOR14	diaphragm	CRS	F	76	T4N2M0	sigmoid	adeno
TOR17	omentum	CRS	M	83	T4N2M1	sigmoid	adeno
TOR22	serosa sigm.	CRS	M	82	T4N2M1	sigmoid	adeno
PM02-1	ascites	PIPAC cycle 1	M	58	pT3N1M0	hepat. flexture	adeno
PM03-1	ascites	PIPAC cycle 1	M	44	unknown	caecum	SRC
PM14-2	ascites	PIPAC cycle 2	F	66	unknown	sigmoid	adeno
CRS, cytoreductive surgery; PIPAC, Pressurized IntraPeritoneal Aerosol Chemotherapy; SRC, Signet ring cell							
